# Modeling and optimization of parallelized immunomagnetic nanopore sorting for surface marker specific isolation of extracellular vesicles from complex media

**DOI:** 10.1038/s41598-023-39746-7

**Published:** 2023-08-16

**Authors:** Andrew A. Lin, Hanfei Shen, Griffin Spychalski, Erica L. Carpenter, David Issadore

**Affiliations:** 1 Department of Bioengineering, University of Pennsylvania, 210 S. 33rd St., Philadelphia, PA 19104 USA; 2 Perelman School of Medicine, University of Pennsylvania, 3400 Civic Center Blvd., Philadelphia, PA 19104 USA

**Keywords:** Biomedical engineering, Biosensors, Magnetic devices, Nanofluidics

## Abstract

The isolation of specific subpopulations of extracellular vesicles (EVs) based on their expression of surface markers poses a significant challenge due to their nanoscale size (< 800 nm), their heterogeneous surface marker expression, and the vast number of background EVs present in clinical specimens (10^10^–10^12^ EVs/mL in blood). Highly parallelized nanomagnetic sorting using track etched magnetic nanopore (TENPO) chips has achieved precise immunospecific sorting with high throughput and resilience to clogging. However, there has not yet been a systematic study of the design parameters that control the trade-offs in throughput, target EV recovery, and ability to discard background EVs in this approach. We combine finite-element simulation and experimental characterization of TENPO chips to elucidate design rules to isolate EV subpopulations from blood. We demonstrate the utility of this approach by reducing device background > 10× relative to prior published designs without sacrificing recovery of the target EVs by selecting pore diameter, number of membranes placed in series, and flow rate. We compare TENPO-isolated EVs to those of gold-standard methods of EV isolation and demonstrate its utility for wide application and modularity by targeting subpopulations of EVs from multiple models of disease including lung cancer, pancreatic cancer, and liver cancer.

## Introduction

Extracellular vesicles (EVs) are nanoscale (< 800 nm) membranous particles containing nucleic acid cargoes and expressing surface proteins which reflect their cells of origin^1^. Because of their multiple cargoes and their ability to circumvent anatomical barriers such as the blood–brain barrier to circulate in peripheral bodily fluids such as blood (10^10^–10^12^ EVs/mL)^2^ and urine (10^10^ EVs/mL)^3^, EVs have become a promising biomarker source for the diagnosis and characterization of multiple cancers^4–9^, as well as in other disease contexts including traumatic brain injury^10^ and infectious disease^11^. Additionally, EVs play a mechanistic role in biological processes such as metastatic seeding^12^ and tumor-immune interactions in cancer^13^, as well as pathologies including traumatic brain injury^14^, autoimmune disease^15^, and cardiac arrest^16^.

Currently, the study of EVs, and their potential for diagnostics and therapeutics, are held back by technology that was not designed to address their unique combination of nanoscale size, complexity, and quantity in bio-specimens. The high concentration of EVs in blood poses a particular challenge for investigators seeking to differentiate a specific EV subpopulation from other EV subpopulations, as well as other non-EV particles such as cell debris in the same size range (non-relevant “background”). Current gold-standard EV isolation methods such as ultracentrifugation, commercial precipitation kits (Thermo Fisher, System Biosciences), and size-exclusion chromatography lack the surface-marker selectivity and throughput to precisely sort EV subpopulations^17^.

Likewise, previously-established methods for surface-marker sorting of cells are unable to measure nanoscale EVs or achieve the throughput to process the large numbers of EVs typically found in clinical samples. For example, processing ~ 10^11^ EVs in 1 mL of blood would not be feasible for high-throughput cell flow cytometry. Typical nanoparticle flow cytometers sort at a rate of ~ 1000 counts/second^18^, requiring ~ 3 years to sort through 1 mL of blood, while even the latest subcellular flow cytometry systems sorting at ~ 60,000 events/second^19^ would require 19 days for 1 mL of blood. This challenge is amplified by the low absolute expression of surface proteins on EVs as compared to cells owing to the dramatically-increased surface area of a ~ 10 µm cell compared to a < 800 nm EV, thus yielding fluorescent signals below the level of detection for commercial flow cytometry systems^20^. In response to this challenge, multiple microfluidic approaches have been developed using EV-sized micro/nanoscale feature sizes to perform precision size-based or surface-marker EV sorting. However, limitations such as the requirement for complex nanofabrication^4,21,22^, low maximum input volumes^23,24^, reliance on a single molecular biomarker target^25^, or low sample throughput^21^ have hindered the applicability of microfluidic size or surface marker EV sorting.

To address the shortcomings of previous generations of microfluidic devices in EV subpopulation isolation, our group has developed Track-Etch magnetic NanoPOre (TENPO) chips to perform the parallelized immunomagnetic sorting of EVs based on their surface proteins^5^. By expanding the parallelization of immunomagnetic sorting to millions of track-etched magnetic nanopores, TENPO is resilient to failure due to clogs in individual pores because a clog causes fluid to redistribute uniformly to the millions of other magnetic nanopores. Moreover, the parallel operation of millions of nanopores increases throughput and sample flow rates, as the individual flow velocity per pore can be kept low based on the high pore density (> 10^7^ pores/cm^2^ for *d* = 600 nm pores^5^, > 10^6^ for *d* = 3 µm pores per Cytiva/Whatman). Lastly, track etching combined with vapor deposition of a bilayer, consisting of a soft magnetic layer of NiFe and a passivation layer of Au, offers inexpensive fabrication of large numbers of precisely-defined magnetic nanopores while bypassing expensive and difficult-to-scale lithography^5^.

**Figure 1:**
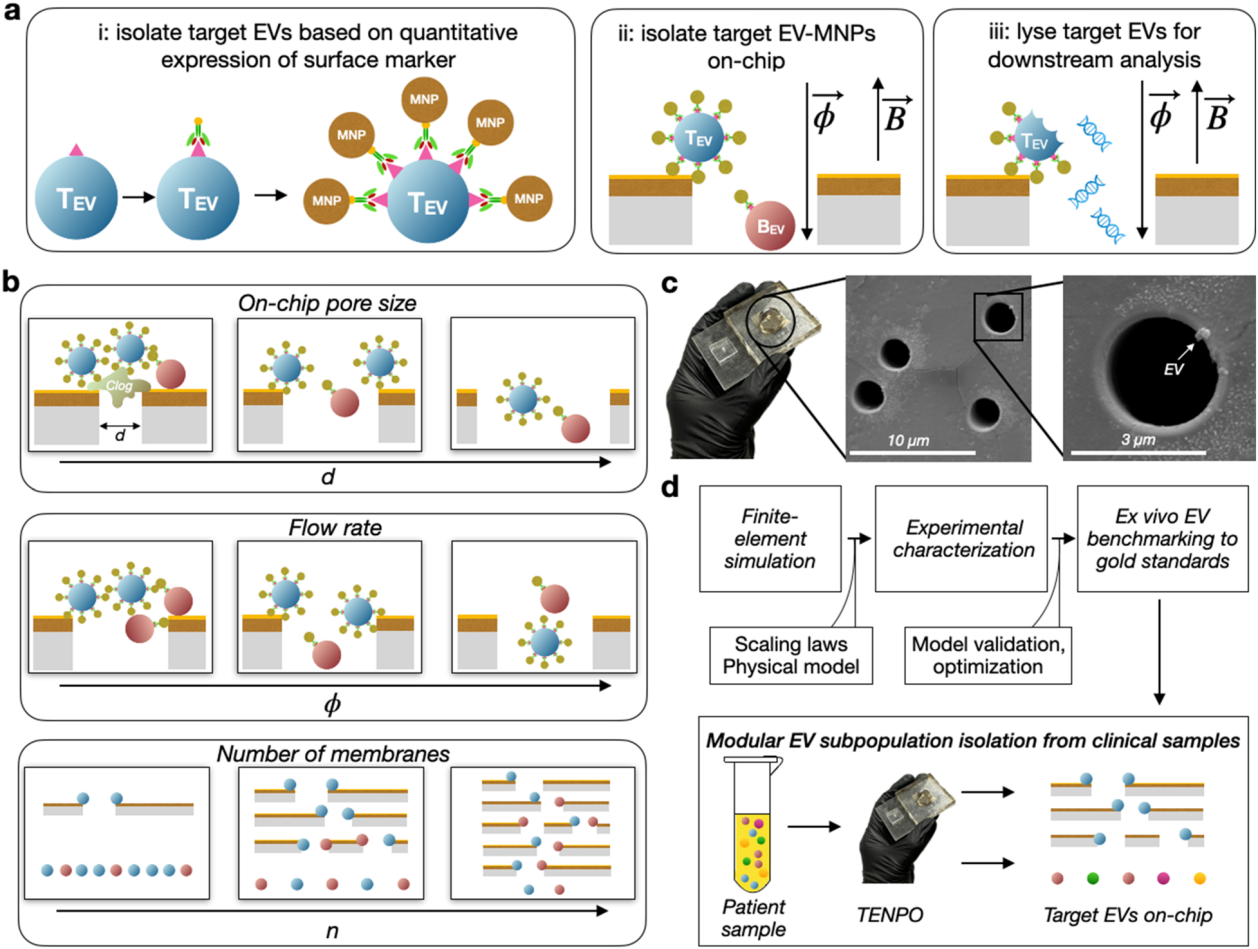
Characterization of TENPO isolation of EV subpopulations. (**A**) Schematic of track-etched magnetic nanopore EV isolation. EVs are first labeled with biotinylated capture antibodies followed by anti-biotin magnetic nanoparticles (50 nm). EV-MNP complexes are magnetically captured as they flow vertically through parallelized magnetic nanopores. (**B**) Illustrations of tradeoffs in TENPO isolation. Adjusting the design parameters—pore diameter d, flow rate *ɸ*, and number of membranes n—results in trade-offs that can be used to tailor TENPO to isolate particular EV subpopulations from clinical specimens. (**C**) Photograph of an assembled TENPO chip (left) and SEM micrographs of the TENPO magnetic nanopores (center and right) with an EV immobilized on-chip (right). (**D**) A schematic of the workflow of this study.

To isolate specific EV subpopulations, EVs are first labeled with biotinylated antibodies specific to surface markers of interest and are then conjugated to 50 nm anti-biotin magnetic nanoparticles (MNPs). EVs that highly express a particular surface marker will thus be strongly labeled with MNPs compared to EVs that weakly express or do not express the surface marker (Fig. 1A, left). EV-MNP complexes are pulled vertically through the magnetic nanopores using a syringe pump, and only EVs which have been tagged with a sufficient number of MNPs will have a sufficiently-strong magnetophoretic force to overcome the drag force of fluid flowing through the pore (Fig. 1A, center). Unlike light-based measurements which are constrained in length scale by the wavelength of light, magnetostatics are not constrained in length scale^5,26^ and are aided by the lack of significant background magnetism in peripheral body fluids such as blood and urine. In TENPO’s workflow, captured EVs are lysed for downstream nucleic acid or protein quantification (Fig. 1A, right), or eluted whole for EV characterization (ex. nanoparticle tracking analysis, NTA). With TENPO, multiple EV subpopulations in a disease (e.g. neuron vs. astrocyte-derived EVs in dementia) can be isolated in a rapid, low-cost chip-based format for downstream cargo analysis.

A key feature of the design of TENPO is that as EVs are captured, or as pores become clogged, it does not significantly impact the performance of the device until > 10% of the magnetic nanopores become occluded. This feature arises because the fluidic resistance of the magnetic nanopores is several orders of magnitude greater than the resistance between pores, and as such when a pore is occluded the flow is distributed not only to its nearest neighbors, but uniformly over the entire 10^7^ pores as if they are in connected in a parallel circuit^27^. Moreover, in many applications of isolating specific sub-populations of EVs, the subpopulation is sparse (ex. ~ 1900 tumor-derived EVs/mL per mm^3^ of tumor volume for highly-shedding tumors)^28^ in comparison to the total number of EVs. Therefore even in a case when TENPO processes 10^11^ EVs in a mL of human plasma, several orders of magnitude less targeted EVs are captured on the TENPO’s ~ 10^7^ magnetic nanopores. Because our device sorts EVs one at a time, in a device that is matched in scale to that of nanoscale EVs, it can sort EVs based on quantitative expression of surface markers, akin to flow cytometry for cell based sorting. This is in contrast to conventional methods that use micrometer-scale sized beads or devices^29^, where EV capture is dictated by a single binding event. Moreover, previous immunoaffinity bead isolation methods have been limited by the requirement that a high proportion of EVs express a given target protein^30^.

Basic evaluation of TENPO’s performance using simple model systems has been previously reported^5,31^. TENPO-based EV isolation from blood has performed both the diagnosis of and metastasis detection in pancreatic cancer with accuracy superior to conventional methods^5,6^, and has been applied in diagnosing traumatic brain injury^10^.

In this manuscript, we describe a systematic study of the design parameters that control the trade-offs in throughput, target EV recovery, and discarding of background EVs/non-EV debris in this approach. We do this by combining finite-element simulation and experimental characterization of TENPO chips to elucidate EV subpopulation isolation design rules (Fig. 1B).

*1)* *Pore diameter* *d* Previous versions of TENPO used a pore size of *d* = 600 nm to bring the size of the device features close to the size scale of EVs^5^. However, nanoscale pore sizes can result in size-based trapping of larger EVs such as microvesicles (~ 100–1000 nm) as well as non-EV background, such as cell debris and apoptotic bodies^32^ (Fig. 1B). Although pre-processing steps can be made more aggressive to remove larger materials, this risks losing target EVs. While increasing *d* reduces size-based trapping to improve purity, it risks increasing the distance that target EV-MNP complexes have to travel for capture on the pore edge, thus diminishing target EV yield.

*2) Flow rate* *ɸ* By tuning the sample flow rate *ɸ* at which sample is flowed through TENPO, the yield for targeted EVs relative to the successful discarding of background EVs can be traded off. With decreasing *ɸ*, fewer bound MNPs are required for a targeted EV to be translated to a pore’s edge and captured. While this increases target EV yield, it also increases the capture of background EVs bound due to non-specific adhesion with few MNPs. With increasing *ɸ*, fewer weakly labeled background EVs are co-isolated at the expense of losing more targeted EVs.

*3) Number of membranes* *n* TENPO membranes can be stacked in series to increase the capture probability of targeted EVs, as each membrane provides an independent capture chance^5^, and thus increases target EV yield. However, increasing *n* leads to increased dead volume on-chip (~ 25 µL per membrane for a 2.5 cm^2^ device) and increased non-specific capture of weakly-tagged background EVs, hence reducing purity.

In this work, we characterize the effect that varying device parameters (*d*, *ɸ*, *n)* has on the performance of surface-marker-selective EV sorting using TENPO (Fig. 1C). To this end, we demonstrate: (1) finite-element simulations to reveal the scaling of device performance with device parameters, (2) experimental validation of these device scaling laws in a model system of pancreatic cancer, (3) benchmarking of our EV isolation to gold standard methods, and (4) the modular isolation of EV subpopulations across three different cancer model systems (Fig. 1D).

## Results

### Modeling immunomagnetic TENPO EV isolation

To identify the scaling laws underlying the performance of TENPO for immunomagnetic sorting, we first conducted a multi-physics finite-element simulation (COMSOL) incorporating a magnetostatic model of the magnetic field gradient, a model of microfluidic flow through magnetic nanopores, and particle tracking simulations of immunomagnetically labeled EVs. Building on previous two-dimensional simulations that took advantage of the radial symmetry of each magnetic nanopore^5^, we performed a three-dimensional simulation. Fluid flow was modeled as flow-velocity-controlled (flow entering the channel through an input plane atop the pore with a defined flow velocity) laminar inflow with a no-slip boundary condition and a periodic flow condition on the simulation edges to simulate a large grid of pores around a given single pore. Magnetophoretic traps formed at the pore’s edge in simulated devices from pore diameter *d* = 600 nm to *d* = 12 µm (SI Fig. 1) in a strong external magnetic field ($|\overrightarrow{B}|$= 0.4 T) producible by an NdFeB magnet (diameter = 1.5 in., height = 0.75 in., K&J Magnetics). We compared the magnetophoretic forces for each pore diameter to the drag force at the pore’s edge at a typical volumetric flow rate of *ɸ* = 2.5 mL/h with a device cross-sectional area of *a* = 2.5 cm^2^, for both strongly-tagged EVs with 15 MNPs bound and weakly-tagged EVs with 1 MNP bound. In this simulation, we found that the vertical magnetophoretic force 100 nm from the pore’s edge is greater than the drag force by ~ 3 orders of magnitude for EVs tagged with 15 MNPs and ~ 2 orders of magnitude for EVs tagged with 1 MNP (SI Fig. 2). Therefore, for all pore diameters considered, the trapping of EVs is determined by whether an EV is magnetophoretically translated to the pore’s edge before it passes through the pore at a velocity dictated by the volumetric flow rate, the pore diameter, and the total number of pores.

**Figure 2:**
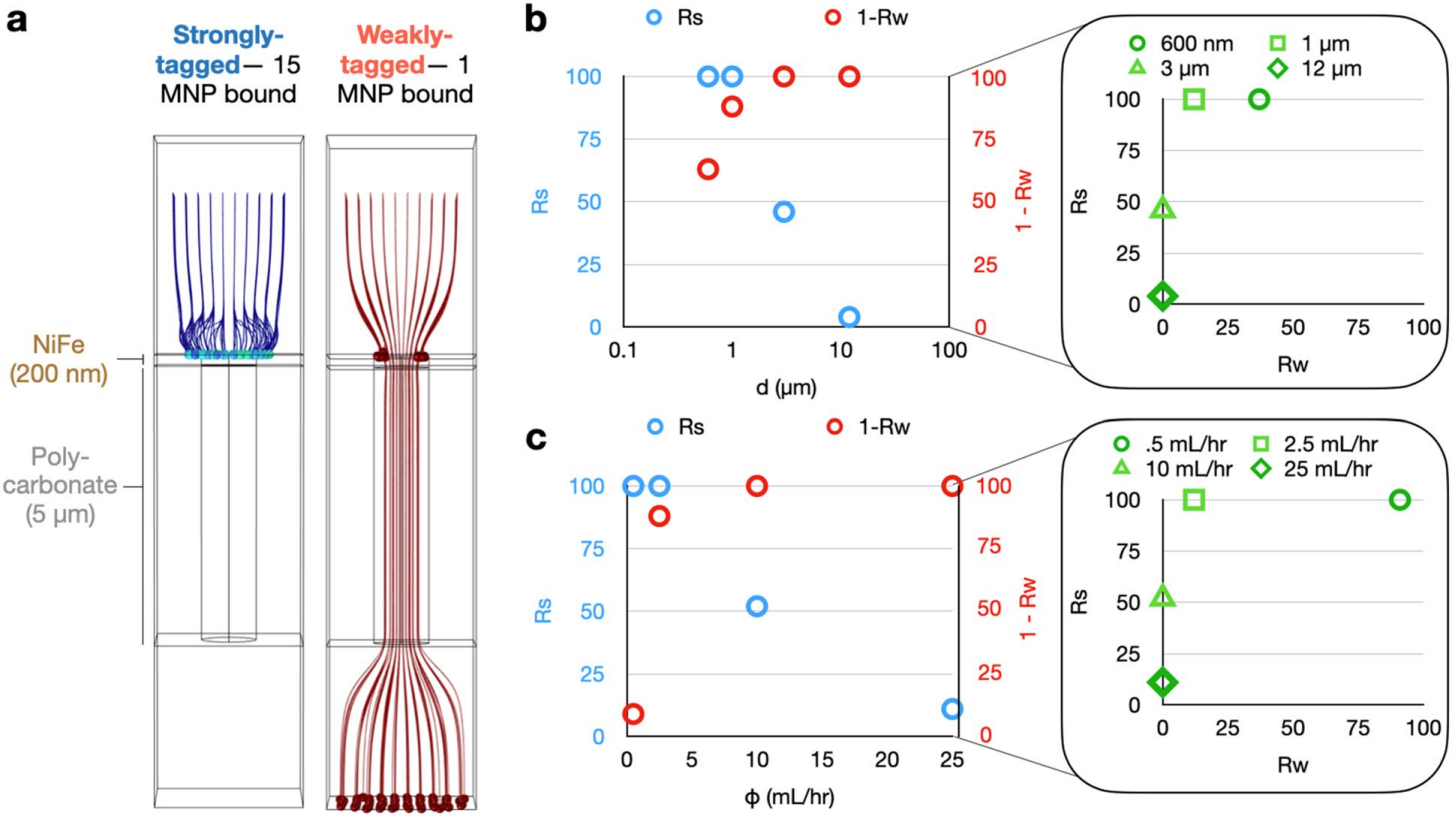
Finite-element simulations to characterize TENPO EV sorting. (**A**) Particle tracking simulations for strongly-tagged versus weakly-tagged EVs through a single magnetic nanopore at an example pore diameter *d* = 1 µm and an example volumetric flow rate* ɸ* = 2.5 mL/h. (**B**) The capture rate of strongly labeled EVs (Rs) and weakly labeled EVs (Rw) versus pore diameter *d* for a volumetric flow rate* ɸ* = 2.5 mL/h. (**C**) The capture rate of strongly labeled EVs and weakly labeled EVs versus volumetric flow rate *ɸ*, for a pore diamete*r d* = 1 µm.

Using the magnetic field and fluid flow simulations described above, we simulated the trajectories of EVs conjugated with MNPs across multiple pore diameters *d*, flow rates *ɸ*, and number of TENPO membranes placed in series *n*. In each simulation we considered the trajectory of 100 EVs that flow through a single magnetic nanopore from a uniform square grid of initial positions with a length twice the pore diameter, and a height 3 µm above the pore. 150 nm diameter EVs were simulated as either being “strongly-tagged” with 15 MNPs bound to an EV or “weakly-tagged” with 1 MNP bound to an EV (Fig. 2A). 50 nm diameter MNPs were modeled based on cross-linked iron oxide (CLIO) ferrite nanoparticles owing to their common usage and characterization in the literature^33,34^.

We first modeled the effects of pore diameter *d* on the performance of TENPO by modeling pore diameters ranging from *d* = 600 nm to 12 microns, assuming a flow rate *ɸ* = 2.5 mL/h, and using particle tracking to quantify their ability to isolate strongly-tagged versus weakly-tagged EVs. We defined the capture rate of strongly-tagged EVs as Rs and the capture rate of weakly-tagged EVs as Rw; 1-Rw was therefore used to evaluate the ability of the device to successfully discard weakly-tagged EVs. For this model system, a pore diameter of *d* = 1 µm yielded the greatest separation between Rs and Rw (Fig. 2B). While Rs decreased as pore diameter increased, 1-Rw increased for *d* = 600 nm and 1 µm before plateauing at 1-Rw = 100% (Rw = 0%) at *d* = 3 and 12 µm. In contrast, Rs was maximal at 100% at *d* = 600 nm and 1 µm before decreasing at *d* = 3 and 12 µm. In this simulation, a pore diameter of 1 µm yielded high Rs = 100% and a moderately high 1-Rw = 88%. However, clinical samples can feature a more complex distribution of MNP conjugation to EVs depending on surface protein expression.

Using our model, we next evaluated the effect on performance of varying the number of TENPO membranes *n* in series. To model multiple TENPOs in series, we performed an iterative simulation whereby EVs that are not captured in a simulation are then simulated passing through a subsequent TENPO with a random initial position, as the placement of pores in each membrane is independent from one another. We considered a magnetic nanopore configuration (*d* = 3 µm at a flow rate *ɸ* = 2.5 mL/h) that had high 1-Rw (ability to discard weakly-tagged vesicles) but low Rs (yield for strongly-tagged vesicles) to evaluate whether Rs could be recovered and 1-Rw preserved with multiple membranes. Here, both Rs and Rw increased with greater *n*. Rs increased faster than Rw up to *n* = 2–3 membranes, which yielded the greatest separation between Rs versus Rw (SI Fig. 3). Adding more than 3 membranes increased Rw with diminishing improvements in Rs.

We then modeled the effect of flow rate *ɸ* on the performance of magnetic nanopores with diameter *d* = 1 µm, chosen for its high Rs and moderately high 1-Rw. For flow rates *ɸ* < 2.5 mL/hr, all strongly-tagged EVs were captured. As the flow rate increased beyond *ɸ* > 2.5 mL/h, Rs decreased as a function of flow rate *ɸ*. At flow rates *ɸ* < 2.5 mL/h, 1-Rw increased as a function of *ɸ,* and beyond *ɸ* > 2.5 mL/h, all weakly targeted EVs were successfully discarded. (Fig. 2C). This model system demonstrates the potential for tuning the tradeoff between Rs and 1-Rw in this model scenario featuring both targeted EVs and off-target EVs with non-specifically bound MNPs.

We also considered the impact of clogging on TENPO's performance. In TENPO, flow distributes uniformly across a large number of magnetic nanopores (*N* = 5.3 × 10^6^ *d* = 3 µm pores in one 2.5 cm^2^ membrane), The average flow velocity through a single pore is V_z_ = *ɸ*/(N*a_*pore*_), where *a*_*pore*_ is the cross-sectional area of a pore. The flow tends to be uniformly distributed across the pores, because the calculated flow resistance between pores is approximately two orders of magnitude lower than the flow resistance through a single *d* = 3 µm pore (SI Fig. 4). Because the nanopores behave as if they are in parallel, blocking a single clogged pore results in its share of the flow being distributed over the other millions of pores operating in parallel on-chip, minimizing its impact on overall performance. In a finite-element simulation of nine pores operating in parallel within a periodic flow condition to simulate a large repeating grid of surrounding pores, we show that the maximal drag force for an EV entering a non-occluded pore is increased by ~ 11% with 1 out of every 9 pores on-grid fully occluded, and is less than doubled (increase of ~ 80%) even with 4 out of every 9 pores fully occluded (SI Fig. 5). With the periodic flow condition, this suggests that the performance of TENPO is not significantly changed even with ~ 10% of the pores fully occluded on-chip.

To evaluate the impact of clogging on a single pore’s performance, we simulated the effect on flow velocity and EV trajectories of strongly-tagged versus weakly-tagged EVs for spherical 400 nm and 800 nm radius clogs on a *d* = 3 µm pore. We observed only limited changes in the velocity profiles and maximum velocities for clogs up to 800 nm (SI Fig. 6). We also performed particle tracking simulations where clogged pores were challenged with n = 100 strongly-tagged versus weakly-tagged EV-MNPs. Rs and Rw did not change for a 400 nm clog, while an 800 nm clog resulted in an increase in Rw but unchanged Rs. Notably, strongly-tagged EVs in the 400 nm and 800 nm clogs accumulated around the location of the clog, while in the 800 nm clog weakly-tagged EVs also accumulated around the location of the clog, indicating increased background trapping (SI Fig. 7).

### Experimental characterization of immunomagnetic TENPO EV isolation

**Figure 3:**
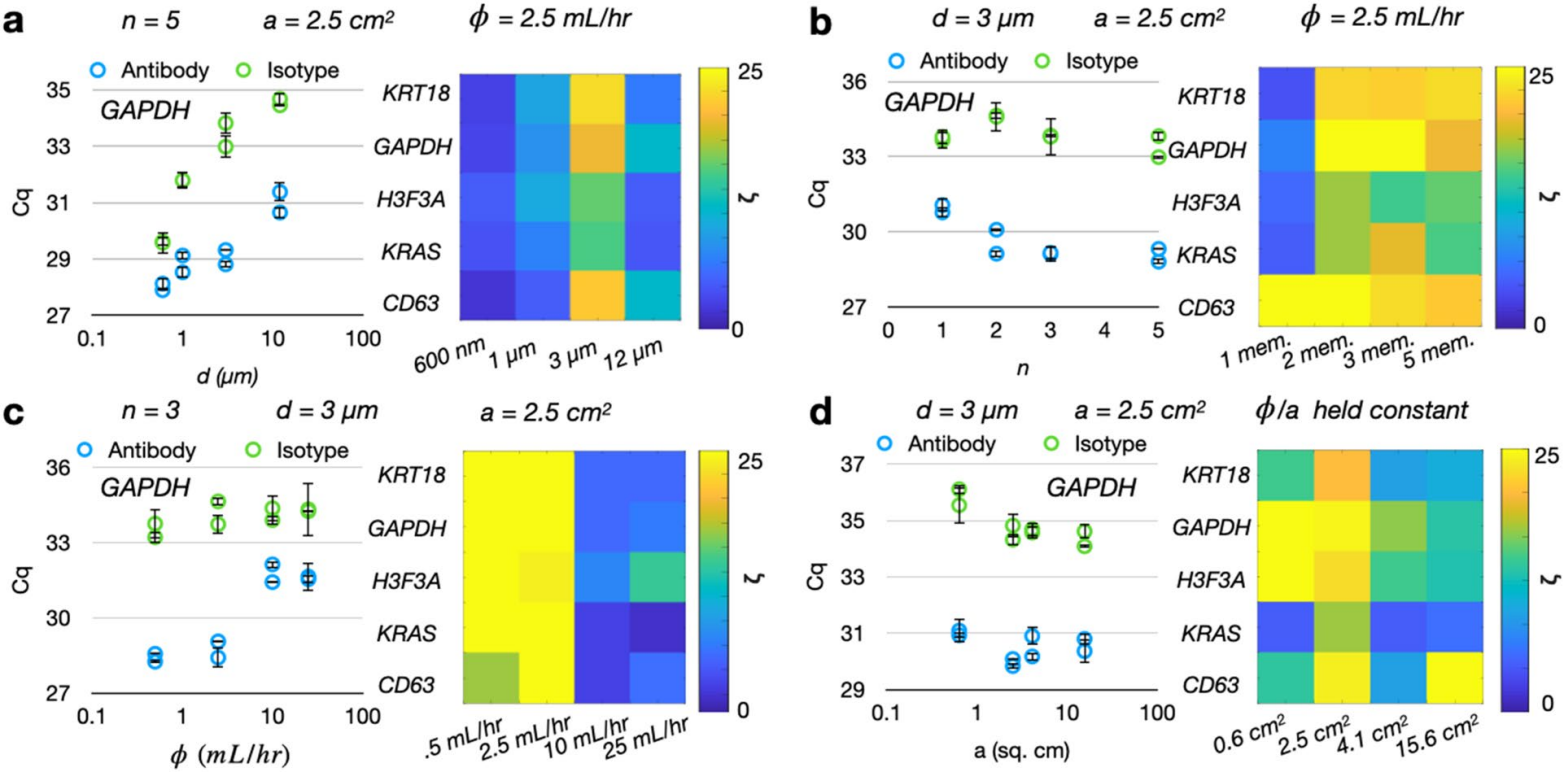
Experimental characterization of TENPO isolation in an in vitro model system of pancreatic cancer. Device parameters which were held constant in the course of the parameter scan are labeled atop each graph set. Each dot represents one device replicate, and error bars are from n = 2 PCR replicates; each condition was ran with two antibody device replicates and two isotype device replicates. Fold-change enrichment ζ was calculated for each condition as 2^(∆Cq) for the ∆Cq between antibody versus isotype devices. (**A**) Isolated EV RNA as a function of pore diameters *d* for antibody-labeled versus isotype-labeled EVs. (**B**) Isolated EV RNA as a function of membrane number *n* for antibody-labeled versus isotype-labeled EVs. (**C**) Isolated EV RNA as a function of flow rate *ɸ* for antibody-labeled versus isotype-labeled EVs. (**D**) Isolated EV RNA as a function of cross-sectional area *a* for antibody-labeled versus isotype-labeled EVs.

The performance of TENPO in immunomagnetically capturing EV subpopulations was characterized experimentally in an in vitro model system of pancreatic cancer. We used TENPO to isolate EVs from pancreatic cancer cell culture media (250 µL, ~ 3.12 × 10^9^ EVs per NTA) spiked into a complex background of 1 mL fetal bovine serum (FBS, 1.03 × 10^10^ EV-sized particles per NTA) meant to provide additional protein and RNA background for challenging the sorting capacity of TENPO. We studied the effect on performance of varying pore diameter *d*, membrane number *n*, flow rate *ɸ*, and cross-sectional membrane area *a*. We also compared on-chip washing flow rates (5 vs. 15 mL/h) (SI Fig. 8) and magnetic field strengths (0.45 vs. 0.34 T) (SI Fig. 9), but found that neither parameter changed our results. For each condition, devices were challenged with either EVs conjugated to pan-EV antibody-labeled MNPs (CD9, CD63, CD81, Biolegend) to represent strongly-tagged EVs or isotype antibody-labeled MNPs (IgGK1, Biolegend) to represent non-specifically labeled background/weakly-tagged EVs. PCR for five nucleic acids (KRT18, GAPDH, H3F3A, KRAS, CD63^5^) was used to measure the levels of captured nucleic acid material, with Cq values being compared between the two conditions. To determine the extent to which nucleic acid levels correlated with EV concentration, we performed a series of spike-in titration experiments where different concentrations of media with pancreatic cancer cell culture EVs were spiked into a constant volume of healthy human plasma. From these spike-in samples, EVs were isolated via ultracentrifugation and then lysed to quantify nucleic acid marker expression via PCR. We demonstrated good correlation between the concentration of spiked pancreatic cancer cell culture EVs and nucleic acid levels (SI Fig. 10) even with the spike-in to complex plasma background. This enables us to validate the trends for EV capture seen in simulation using the changes in nucleic acid levels between device conditions as an approximate readout of EV capture. For each parameter, the results are discussed below:

*Pore diameter* *d* We evaluated TENPO chips with *d* = 600 nm, 1 µm, 3 µm, and 12 µm. The total number of membranes (*n* = 5), flow rate (*ɸ* = 2.5 mL/h), and cross-sectional area of the device (*a* = 2.5 cm^2^) were held constant. The quantity of EV RNA isolated using a pan-EV cocktail of CD9, CD63, and CD81 versus an isotype antibody control was characterized. The EV-derived RNA isolated via the pan-EV cocktail remained constant for pore diameters *d* = 3 µm and below and decreased for larger pore diameters (Fig. 3A, SI Fig. 11), agreeing with the trend predicted by simulation (Fig. 2B). Likewise, the EV RNA isolated using the isotype control was highest for the lower pore diameters at *d* = 600 nm before decreasing to a minimum at *d* = 3 µm and *d* = 12 µm (Fig. 3A, SI Fig. 11), agreeing with the trend predicted by simulation (Fig. 2B). To consider the differences in the total porous area between the *d* = 600 nm membranes as compared to the larger-pore-diameter membranes, we also performed a set of experiments where we scaled the flow rates for porous area to keep per-pore flow velocity constant. In this experiment, the same trend was observed as found without keeping velocity constant (SI Fig. 12).

*Membrane number* *n:* We evaluated the effect of stacking multiple membranes in series on the capture of antibody-labeled versus isotype-labeled EVs. In these experiments, the pore diameter *d* = 3 µm, flow rate *ɸ* = 2.5 mL/h, and device cross-sectional area *a* = 2.5 cm^2^ were held constant. Experimentally, we observed an increase in recovered target EV RNA as the number of membranes was increased (Fig. 3B, SI Fig. 11), and this trend was similar to the predicted trend in simulation where the amount of targeted EVs captured increased as the number of membranes increased to *n* = 3, but plateaued beyond that (SI Fig. 3). In contrast to the predicted increase in background from simulation, the experimental measurement of isotype-labeled EV nucleic acids remained constant across *n* = 1 to *n* = 5 membranes (Fig. 3B, SI Fig. 11). We hypothesize that this difference may be due to the Cq values for the isotype background being closer to the limit of detection of our PCR assays (Cq values of > 33).

*Flow rate* *ɸ* We considered four different flow rates: *ɸ* = 0.5 mL/h, 2.5 mL/h, 10 mL/h, and 25 mL/h, while keeping the pore diameter *d* = 3 µm, membrane number *n* = 3, and device cross-sectional area *a* = 2.5 cm^2^ constant. As predicted by simulation, we observed a decrease in recovered target EV RNA as flow rate *ɸ* increased between* ɸ* = 2.5 mL/h and *ɸ* = 10 mL/h. However, unlike in simulation, we then observed that the recovered target EV RNA plateaued for flow rates *ɸ* > 10 mL/h rather than decreasing (Fig. 3C, SI Fig. 11). This difference may be due to differences in magnetic labeling where EVs with greater than 15 MNPs are still captured at high flow rates. We observed no change in isotype-labeled EV RNA at any of the flow rates, in contrast to the predicted decrease in background on simulation. As in the membrane number scan, we hypothesize that the isotype background Cq values are closer to the limit of detection of our PCR assays. We observed the greatest difference between the antibody-labeled versus isotype-labeled EVs at a flow rate of *ɸ* = 2.5 mL/h (Fig. 3C, SI Fig. 11).

*Cross-sectional area:* We tested four different cross-sectional areas *a* (designs for all devices shown in SI Fig. 13) for the TENPO devices, keeping the pore diameter constant at *d* = 3 µm. To keep per-pore flow velocity constant, we compensated for the changes in total open area by changing the sample and wash flow rates. We observed a small (< 1 ∆Cq) increase in both antibody-labeled EV signal and isotype-labeled EV signal which scaled with increasing *a* (Fig. 3D, SI Fig. 11). We also observed a decrease in the fold-change enrichment between antibody-labeled versus isotype-labeled EV nucleic acid signal with increasing *a* (Fig. 3D, SI Fig. 11).

EVs are known to be heterogeneous in their size and their surface marker expression and our results reflected that. The RNA cargo isolated from our model system using the “pan-EV” (CD9, CD63, CD81) markers was significantly (*p* < .05) greater than the isotype antibody control. Significant differences versus the isotype antibody control were also observed for at least one nucleic acid marker for CD9, CD63, and CD81 individually (*p* < .05). Of the three individual markers, CD9 yielded more RNA compared to either CD63 and CD81 (SI Fig. 14), which was consistent with ELISA (SI Fig. 15). We also quantified run-to-run variability of our TENPO isolation. We observed a standard deviation < 1 Cq (similar to our PCR replicate variation) in a comparison of pan-EV EV isolations run on different days (six antibody and six isotype replicates) (SI Fig. 16). A one-way ANOVA revealed no significant differences for each marker within any of the antibody or isotype replicates for KRT18 and H3F3A (*p* > .05), while for GAPDH there was a significant difference between the antibody replicates (*p* = .014) but not the isotype replicates (*p* > .05).

### Benchmarking TENPO to commercial gold standards and demonstrating modular subpopulation isolation

**Figure 4:**
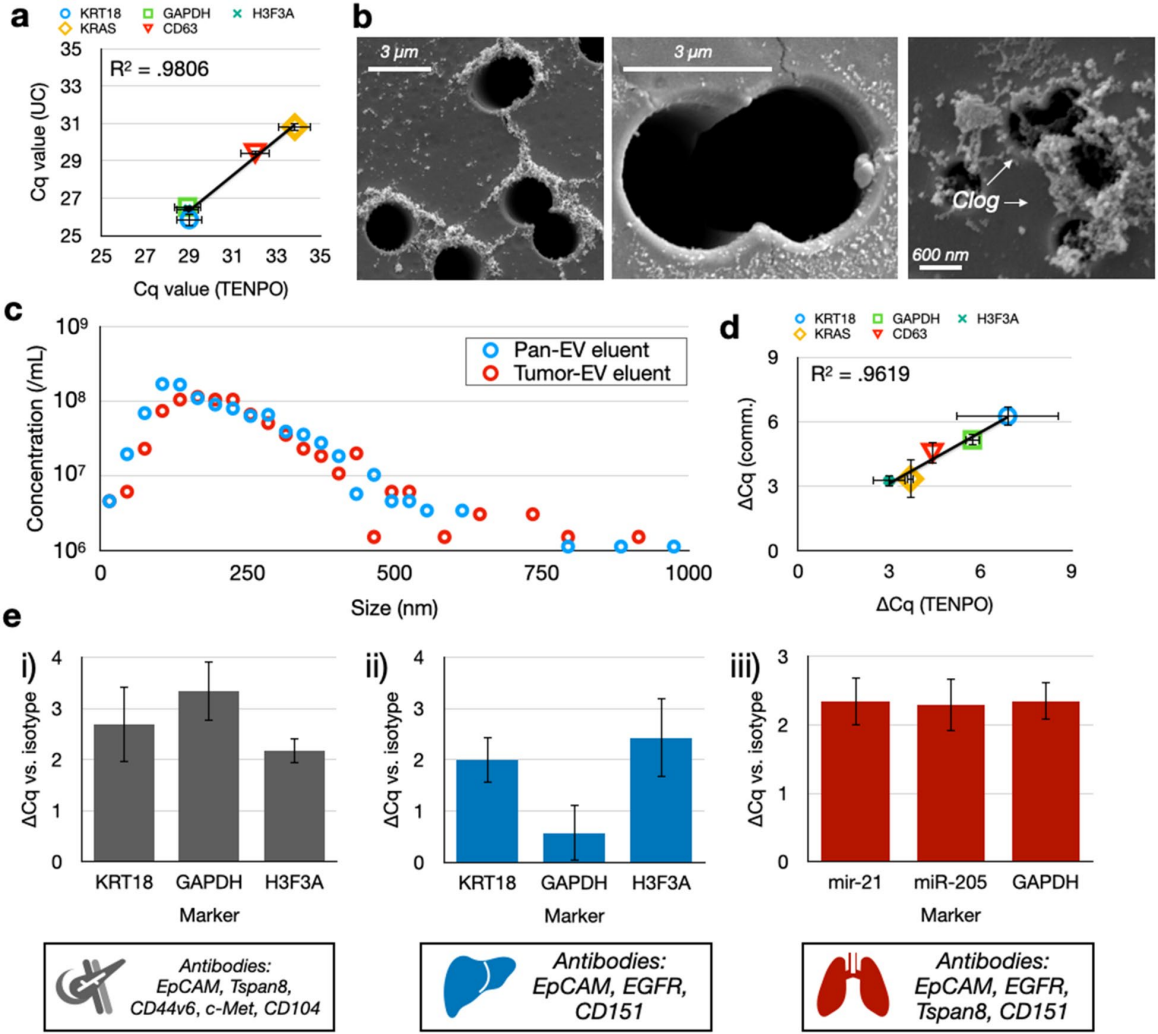
In vitro benchmarking of TENPO to gold standard technologies and in different biological systems. (**A**) Correlation of nucleic acid cargoes between TENPO versus UC. Each point corresponds to a nucleic acid marker measured in the CD9/CD63/CD81 + EVs isolated using TENPO compared to the same nucleic acid markers measured in EVs isolated using UC. Error bars from n = 2 device/prep replicates. (**B**) SEM micrographs of EVs captured on TENPO. The left and middle micrographs show a TENPO with *d* = 3 µm magnetic nanopores. The right micrograph shows a clogged TENPO with *d* = 600 nm magnetic nanopores. (**C**) Size distributions of EVs captured by TENPO and eluted for measurement by NTA. (D) Comparison of ∆Cq between cancer cell culture media spiked into plasma versus control cell culture media spiked into plasma for pan-EV TENPO vs. a commercial pan-EV kit. Error bars from n = 2 device/prep replicates (two case, two control) using propagation of error. (**E**) Comparison of ∆Cq between antibody-labeled versus isotype-control-labeled EVs in three different model systems of cancer. Error bars from n = 2 device/prep replicates (two antibody devices, two isotype devices) using propagation of error.

We benchmarked TENPO EV isolation versus conventional methods. Based on the results of the prior section, we chose to use *n* = 3-membrane, *d* = 3 µm, *a* = 2.5 cm^2^ TENPO chips run at a flow rate *ɸ* = 2.5 mL/h. We found that the RNA cargo (KRT18, GAPDH, H3F3A, KRAS, CD63) isolated from cell culture media using TENPO (CD9, CD63, CD81) correlated well on PCR (R^2^ = .98) with the cargo isolated using UC (Fig. 4A). We hypothesize that additional non-EV background and cellular debris was responsible for the lower Cq values (i.e. additional nucleic acid signal) seen for the UC-isolated EVs as compared to TENPO, which used pan-EV markers whose expression is not necessarily universal across all EVs across all cell lines^35^. The additional retention of potential non-EV background on UC is supported by subsequent experiments which challenged UC versus TENPO with isolating EVs from healthy human plasma. Here, TENPO achieved a much higher depletion of albumin, a conventional measure of non-EV background and relative EV purity^36^, compared to UC (36× vs. 4.5×) (SI Fig. 17). In contrast, the total number of EVs isolated, size distribution (SI Fig. 18) and total protein in the EV isolate (SI Fig. 17) were consistent between UC and TENPO. SEM of TENPO with *d* = 3 µm magnetic nanopores validated that the device captured EVs labeled with MNPs at the pore’s edge, while SEM of TENPOs with *d* = 600 nm magnetic nanopores identified greater clogging (Fig. 4B). We also characterized the sizes of whole EVs eluted off TENPO with either the pan-EV pulldown described above or a five-marker tumor pulldown (EpCAM, CD44v6, Tspan8, CD104, c-Met) described in our previously-published work^6^. We quantified the eluted EV isolate and found that the size of EVs isolated using TENPO matched EVs isolated using UC and results from our prior work on TENPO (Fig. 4C) (SI Fig. 18)^5,37^.

We also compared the ability of TENPO to isolate EV nucleic acid cargoes from complex background representative of human patient samples versus a commercial pan-exosome isolation kit (Fujifilm). We challenged TENPO and the commercial affinity kit with two different sample types. We spiked either 250 µL pancreatic cancer cell culture media or 250 µL control cell culture media (media not exposed to pancreatic cancer cells) into 750 µL healthy human plasma. We chose a pan-EV pulldown for this experiment to best match existing pan-EV commercial platforms. The difference in nucleic acid cargoes from the EVs isolated from each sample by each method showed a close correlation on PCR (R^2^ = .96) between TENPO versus the commercial affinity kit (Fig. 4D).

Lastly, we demonstrated the modularity of TENPO across multiple disease contexts. Using cell culture models of pancreatic cancer, liver cancer, and lung cancer, we used tumor-specific antibody panels derived from the literature to isolate EVs from cell culture media spiked in FBS. We first compared the isolation of EVs for each antibody panel to isotype controls and observed antibody-mediated enrichment of EV-derived nucleic acids for all three cancer antibody pulldowns (Fig. 4E). We then challenged TENPO with spike-in samples which consisted of “cancer” spike-ins with conditioned cancer cell culture media spiked into healthy human plasma versus “healthy” controls with healthy human plasma spiked with an equivalent volume of non-conditioned cell culture media. Across all three cancers, TENPO demonstrated strong ∆Cqs between the cancer versus healthy spike-in plasma samples (SI Fig. 19). Taken together, these results demonstrate the modularity of TENPO, which can use commercially-available antibodies for a wide variety of targets for EV subpopulation isolation from complex samples.

## Discussion

In this work, we present the modeling and experimental characterization of the parameter space controlling the performance of TENPO in parallelized immunomagnetic nanopore EV sorting. We demonstrate that by controlling the pore diameter *d*, the flow rate *ɸ*, and the number of membranes in series *n*, the recovery of targeted EV subpopulations can be precisely traded off with non-specific background material capture from clinical specimens. We experimentally validated the precise sorting of TENPO and its underlying scaling laws using a model system of pancreatic cancer. We demonstrated the modularity of this approach across multiple model systems of cancer against multiple controls and in commercial as well as conventional methods.

While our finite-element simulations of TENPO captured useful trends in the performance of TENPO, it has several limitations. In the model used for this work, we considered EVs bound to 15 MNPs versus EVs bound to 1 MNP. In practice, the number of MNPs per EV will present as a unique distribution for each application. Moreover, a nucleic-acid signal from a target EV is not necessarily exclusive to a single EV subpopulation, and may be contained in background EVs due to EV heterogeneity and nucleic acids having roles in different biological pathways. Additionally, EVs from the same target cell type can feature heterogeneous expression of specific nucleic acids, thus making the quantification of absolute counts of EVs expressing specific surface markers using nucleic acid markers challenging. As a result, we focused primarily on relative changes between different device operating conditions when comparing our experimental data to our simulation data. TENPO could be biased towards larger EVs which can bind more magnetic nanoparticles via their increased surface area. This could be optimized by using smaller nanoparticles for labeling compared to the 50 nm commercially-available nanoparticles used in this study. Lastly, the simulation results presented here analyze parameters singly, holding all other parameters constant. This assumes that each parameter has an independent effect on device operation, with no interaction between parameters. Further performance improvements could be made if such interactions were characterized, potentially using automated design-of-experiments algorithms^38^.

For EV-derived biomarkers to reach clinical application, techniques with manufacturability and throughput suitable for large numbers (n > 1000) of clinical samples are required. By virtue of its low construction/operation cost (cost for one pan-EV prototype TENPO assay =  ~ $35; $12 material/fabrication^5^, $5 antibody, $18 beads) and compatibility with roll-to-roll manufacturing, TENPO could be scaled up to fast chip manufacturing while also having the throughput for large clinical cohorts. The fabrication cost of TENPO is invariant to pore diameter, unlike most microfluidic approaches which rely on lithographic fabrication. Previous work in our group with TENPO using 600 nm pores to isolate EV subpopulations was able to yield clinically-relevant diagnostic information in n = 204 pancreatic cancer samples^6^ as well as in n = 96 traumatic brain injury samples^10^. In these cases, TENPO using 600 nm pores was able to distinguish biological nucleic acid signals from background, which can be improved even further with the 10× improvement in specificity versus isotype background suggested in this manuscript.

The development of TENPO to isolate EV subpopulations from clinical specimens offers new opportunities in biomarker development and understanding EV biology. As EV-based diagnostics move towards clinical application, the literature has shown how EV heterogeneity drives cancer biology^39^, influences diagnostics^40,41^, and modulates cargoes in different EV types^42^. Using immunomagnetic isolation via specific surface markers, TENPO can take advantage of EV heterogeneity by sorting distinct EV subpopulations from a single patient sample. Analytes such as platelets^43^, white blood cells^44^, and circulating tumor cells^45^ have shown promise in diagnosing cancer, and their EV subpopulations could offer unique cancer biomarkers. To take advantage of the diversity of cells and their EVs, it is important to develop accurate and high-throughput EV sorting technologies. By combining the specificity of immunomagnetic labeling with the improved yield and high throughput of parallelization, TENPO offers the potential for the rapid isolation of EV-subpopulation-derived biomarkers towards both clinical application and biological investigation.

## Methods

### Finite-element simulations

Finite-element simulations were conducted via COMSOL 5.3 using the *Magnetic Fields, No Currents* module for the magnetic field simulation and the *Laminar Flow* module for the flow simulation. The results from the magnetic field and laminar flow simulation were then combined using the *Particle Tracking for Fluid Flow* module to perform particle tracking simulations. Similar to our previous work^37^, the relative permeabilities of the material layers on TENPO in COMSOL were set to approximate the saturation magnetization values of the 200 nm layer of NiFe in the device (~ 7900 Gauss)^46^ at an input field of 341,000 A/m^37^. A 200 nm diameter sphere was chosen to model EV-MNP complexes because its volume was equivalent to a 150 nm diameter EV + 15 MNPs with a 50 nm diameter. The relative permeability of the fluid and a 5 µm backing polycarbonate layer was set to unity. Magnetophoretic force numbers were determined using the extracted values from COMSOL simulations combined with a formulation from^47^. For these MNPs, a saturation magnetization of 95 Am^2^/kg was used, which is within the range of 80–100 Am^2^/kg reported for magnetite and maghemite^48^. We simulated MNPs with a 6 nm diameter iron oxide core in a shell of non-magnetic material with a total hydrodynamic diameter of 50 nm. Differential tagging of EVs on-chip was simulated via adjusting the relative permeability of the EV-MNP complex simulated in COMSOL for the strongly-tagged (15 MNPs bound, µ = 1.00089) versus weakly-tagged (1 MNP bound, µ = 1.00009) conditions in a formulation adapted from^37^. The maximum number of 15 MNPs was also chosen via a derivation as well as SEM data validating that EVs could bind 15 MNPs from^37^. For the scan of pore diameters at 2.5 mL/h, we adjusted the pore density for each pore diameter such that average input vertical fluid velocity through the pore remained constant. For the scan of clog sizes, clogs were modeled as spheres placed on the edge of a pore with the sphere’s diameter protruding into the lumen of the pore and a constant input vertical flow velocity.

### Cell culture media sample preparation

Cell culture media was prepared per a protocol detailed in our previous work, which is reproduced here in full via^37^. The Panc1 (pancreatic cancer), SNU449 (liver cancer), and the lung cancer cell lines H322, H358, H1975, H460, H1299, and H1264 were used; all cell lines listed were purchased from ATCC. Media was cultured in Dulbecco’s Modified Eagle’s Medium (Corning), 10% Fetal Bovine Serum (Sigma-Aldrich) and 50 mg/mL Gentamicin (Gibco) in 75 cm^2^ culture flasks. The culture was maintained in a 37 °C incubator with a 5% CO_2_ atmosphere. The media was renewed two to three times per week, and the cells were sub-cultured at a ratio of 1:3 or 1:4 when 80–90% confluence was reached. To prepare the conditioned media, the cells were transferred to 150 mm × 20 mm tissue culture dishes and seeded at a concentration of 1.3 × 10^7^ cells per dish. The cells were cultured for 5 days in DMEM complete growth media prepared with exosome-depleted FBS. After the 5-day incubation period, the conditioned media was collected and underwent a two-spin centrifugation process to remove large cell debris: the media was spun at 1600×*g* for 10 min (swinging bucket, brake off); the supernatant was isolated and centrifuged at 3000×*g* for 10 min (swinging bucket, brake off). The conditioned media was then aliquoted at 1 mL and stored at − 80 °C for future use.

### Conventional, commercial EV isolation

#### UC

Both cell culture media and plasma samples were first triple-spun at 1600×*g* for 10 min. followed by two 3000×*g* spins for 10 min. to remove cellular debris. Samples were then processed via a 120,000×*g* spin for 2 h at 4 °C per^49^ in a Beckman-Coulter Optima XL-100 K ultracentrifuge using a Beckman-Coulter SW28 rotor at the Extracellular Vesicle Core at the University of Pennsylvania.

#### Commercial kit

The Fujifilm MagCapture™ Exosome Isolation Kit PS Ver.2 was used following the manufacturer instructions.

### EV subpopulation isolation

#### EV, bead conjugation

For all pulldowns, EV-containing samples were first incubated with the antibodies listed below for 20 min on a nutating mixer. 50 µL of anti-biotin ultra-pure magnetic nanoparticles (Miltenyi) were then added to bind to the biotinylated antibodies which were in turn bound to target EVs. Mixing proceeded on a nutating mixer for an additional 20 min before EV-MNP complexes were flowed on-chip.

#### Device operation

Devices were blocked with 700 µL Pluronic F-127 (1% in DI water) for 1 h at a flow rate of ɸ = 0.5 mL/h before a 1 mL PBS wash at ɸ = 15 mL/hr before the addition of sample at the flow rates specified in the manuscript. Following sample flow, washing was conducted with three 700 µL PBS washes at a wash rate of ɸ = 15 mL/h unless otherwise specified.

#### Pan-EV

CD9, CD63, and CD81 (Biolegend) were used at an antibody concentration of 1.25 µg Ab/mL sample. All three antibodies shared the same IgGK1 control (Biolegend).

#### Pancreatic cancer (tumor pulldown)

The following antibodies were used, adding 1 µL of each antibody to our sample per our previously published work^6^: EpCAM (Biolegend), CD104 (Thermo Fisher), c-Met (Thermo Fisher), CD44v6 (Thermo Fisher), Tspan8 (Miltenyi). The isotype controls used were the Biolegend IgGk2b (to match EpCAM, CD104) and IgGK1 (c-Met, CD44v6) alongside the Miltenyi REA Isotype control (to match Tspan8). Samples were run at a flow rate of ɸ = 2.5 mL/h.

#### Liver cancer

The following antibodies were used at a concentration of 1 µg Ab/mL sample: EpCAM (Biolegend), CD151 (Miltenyi), and EGFR (Thermo Fisher). The isotype controls used were the Biolegend IgGk2b (to match EpCAM), Miltenyi REA Isotype control (to match CD151), and the Biolegend IgGK1 (to match EGFR). Notably, the liver cancer EV subpopulation isolation required filtering with a 0.45 µm filter unit (GE Whatman) to achieve strong specificity versus isotype control. Samples were run at a flow rate of ɸ = 2.5 mL/h.

#### Lung cancer

The following antibodies were used at a concentration of 1 µg Ab/mL sample: EpCAM (Biolegend), EGFR (Novus, biotinylated via the Miltenyi one-step biotinylation kit), CD151 (Miltenyi), and Tspan8 (Miltenyi). The isotype controls used were the Biolegend IgGk2b (to match EpCAM), Miltenyi REA Isotype control (to match CD151 and Tspan8), and Novus Rabbit IgG (to match EGFR). Samples were run at a flow rate of **ɸ = **2.5 mL/h.

#### Cancer versus healthy spike-in experiment

Spike-in models of cancer versus healthy patients were made. The “cancer” samples were made by adding media containing predetermined amounts of EVs (0.25 mL—>  ~ 3 × 10^9^ EVs for pancreatic cancer, 1 mL—>  ~ 9 × 10^10^ for lung cancer, 1 mL—>  ~ 9 × 10^8^ for liver cancer) into healthy human plasma (0.75 mL healthy human plasma for pancreatic cancer, 0.25 mL healthy human plasma for lung and liver cancer; plasma has an EV concentration of 2 × 10^12^ EVs/mL as measured via NTA). Equivalent volumes of non-conditioned clean culture media (media not exposed to cancer cells) were added to the volumes of healthy human plasma stated for each cancer media type to make the “healthy” samples.

### Nucleic acid quantification

#### Lysis/RNA isolation

For the nucleic acid characterization experiments, EVs were either lysed post-isolation for UC/commercial kits or on-chip for TENPO in 700 µL QIAzol (Qiagen) following manufacturer instructions. A commercial RNA isolation kit (miRNEasy Mini Kit) was used, and isolated RNA was eluted in 20 µL RNase-free water before storage at − 80 °C or immediate usage for analysis.

#### PCR

The PrimeScript RT Reagent kit (Takara) was used to convert RNA to cDNA following manufacturer instructions, and said cDNA was then run using SYBR-based qPCR (Bio-Rad SYBR Green Master Mix) via manufacturer instructions. For measurement of miRNA markers, the miRCury LNA RT kit (Qiagen) was used to convert RNA to cDNA, with the following PCR taking place via the miRCury LNA SYBR Green PCR kit (Qiagen) via manufacturer instructions. Cycle threshold (Ct) values were measured on a Bio-Rad CFX384 C1000 thermocycler, with thresholds being set automatically by the instrument at ten times the standard deviation of the baseline fluorescence. mRNA primers were used based on previous work in our group^5,6,37^.

#### EV elution

For EV elution, the isolation proceeded as described previously above until after the wash steps with PBS. EVs captured on-TENPO were incubated with 1 mL IgG Elution Buffer (Thermo Fisher) while on-magnet for 10 min at a flow rate of *ɸ* = 0.5 mL/h. IgG Elution Buffer containing EVs was then washed off-chip at *ɸ* = 2.5 mL/h before neutralization with 100 µL of 1 M Tris–HCl per manufacturer instructions.

### Nanoparticle tracking analysis

Nanoparticle tracking analysis was performed using a ZetaView PMX220 Twin at the Extracellular Vesicle Core at the University of Pennsylvania. All dilutions were conducted in DI water and used to adjust the final concentrations reported in the manuscript. Particles measured on NTA were considered as EV-sized if they fell within a range between 45 and 255 nm.

### Device fabrication

Track-etch membranes were coated with nickel–iron per a protocol detailed in our previous work^5^, and were either coated at the Singh Center for Nanotechology (figure panels 3A, 3B, SI Fig. 8, SI Fig. 9, SI Fig. 12) at the University of Pennsylvania or via a commercial supplier (Chip Diagnostics) for all other experiments described. After the selection of different track-etch membrane materials, the enclosing TENPO devices were assembled as previously reported via laser-cut (VLS 2.3) Mylar and double-sided sticky tape in^5,37^ with the use of the patterns described in SI Fig. 13.

### Whole-EV ELISA

We used a whole-EV ELISA which we previously reported in^37^ to characterize surface proteins on the EVs isolated by precipitation kit from pancreatic cancer cell culture media. The protocol for said ELISA is reproduced here; between each step, we performed three washes (five washes after the addition of HRP-streptavidin) in a washing buffer consisting of PBS with 0.05% Tween 20. EVs which were pre-isolated via a commercial precipitation kit (Thermo Fisher) were first immobilized using an alkaline coating buffer (0.455 g Na_2_CO3, 0.90 g NaHCO3, 150 mL DI water) on a high-binding 96-well plate (Greiner) for 2 h followed by an overnight 4 degrees C blocking step in Thermo Fisher Superblock (PBS) Blocking Buffer. EVs were then incubated with biotinylated detection antibodies for 1 h at a concentration of 2 µg/mL in each 200 µL 96-plate well before the addition of 10 µL diluted 1:16,000 HRP-streptavidin (Thermo Fisher) and the reading of fluorescent signal on a plate reader (Tecan Infinite M200).

### EV characterization methods: on-chip EV SEM

Scanning electron microscope experiments were carried out at the Cell and Developmental Biology Microscopy Core (Perelman School of Medicine, University of Pennsylvania) via a protocol previously reported by our group^37^ which is reproduced here. Following addition of sample and completion of sample flowing and washing steps, EV-MNP complexes were immobilized on-chip. Samples were washed three times with 50 mM Na-cacodylate buffer, fixed for 2 h with 2% glutaraldehyde in 50 mM Na-cacodylate buffer (pH 7.3), and dehydrated in a graded series of ethanol concentrations through 100% over a period of 1.5 h. Dehydration in 100% ethanol was done three times. After the 100% ethanol step, dehydrated samples were incubated for 20 min in 50% HMDS in ethanol followed by three changes of 100% HMDS (Sigma-Aldrich Co.) and followed by overnight air-drying as described previously. Then samples were mounted on stubs and sputter coated with gold palladium. Specimens were observed and photographed using a Quanta 250 FEG scanning electron microscope (FEI, Hillsboro, OR, USA) at 10 kV accelerating voltage^50^.

### Protein, albumin quantification

EV protein content was measured via a Qubit 4 Fluorometer. For quantifying albumin contamination, a commercial albumin ELISA kit (Thermo Fisher) was used) on whole EVs isolated from 1 mL of human plasma using either TENPO versus ultracentrifugation. A standard curve was fitted via^51^ (SI Fig. 20). Albumin depletion was calculated relative to a literature-derived value for albumin concentration in human plasma of 4 g/dL^52^; at a dilution of 1:500,000, the original input plasma (Zen-Bio) yielded a fluorescence result well beyond the logistic calibration curve limit of 1200 ng/mL of the ELISA.

### Ethical disclosure

Healthy human plasma was purchased from Zen-Bio, a commercial supplier of cell-based reagents.

### Supplementary Information

Supplementary Figures.

## Data Availability

The datasets generated during and/or analyzed during the current study are available from the corresponding author on reasonable request.
