# Supplementary material for: Modeling and optimization of parallelized immunomagnetic nanopore sorting for surface marker specific isolation of extracellular vesicles from complex media
Source: Sci Rep. 2023 Aug 16;13:13292. doi: 10.1038/s41598-023-39746-7 (PMC10432479; doi:10.1038/s41598-023-39746-7)

**Supplementary Information**

SI fig. 1: Simulation of magnetic field strengths at four different pore diameters alongside magnetophoretic force linecuts (radial component). All linecuts are taken for a line 100 nm above the pore surface extending 0.5x the pore diameter in each direction. Magnetic field magnitude map at *d* = 600 nm provided as example. Greyscale lines indicate location of pore edges (vertical) and zero force (horizontal).
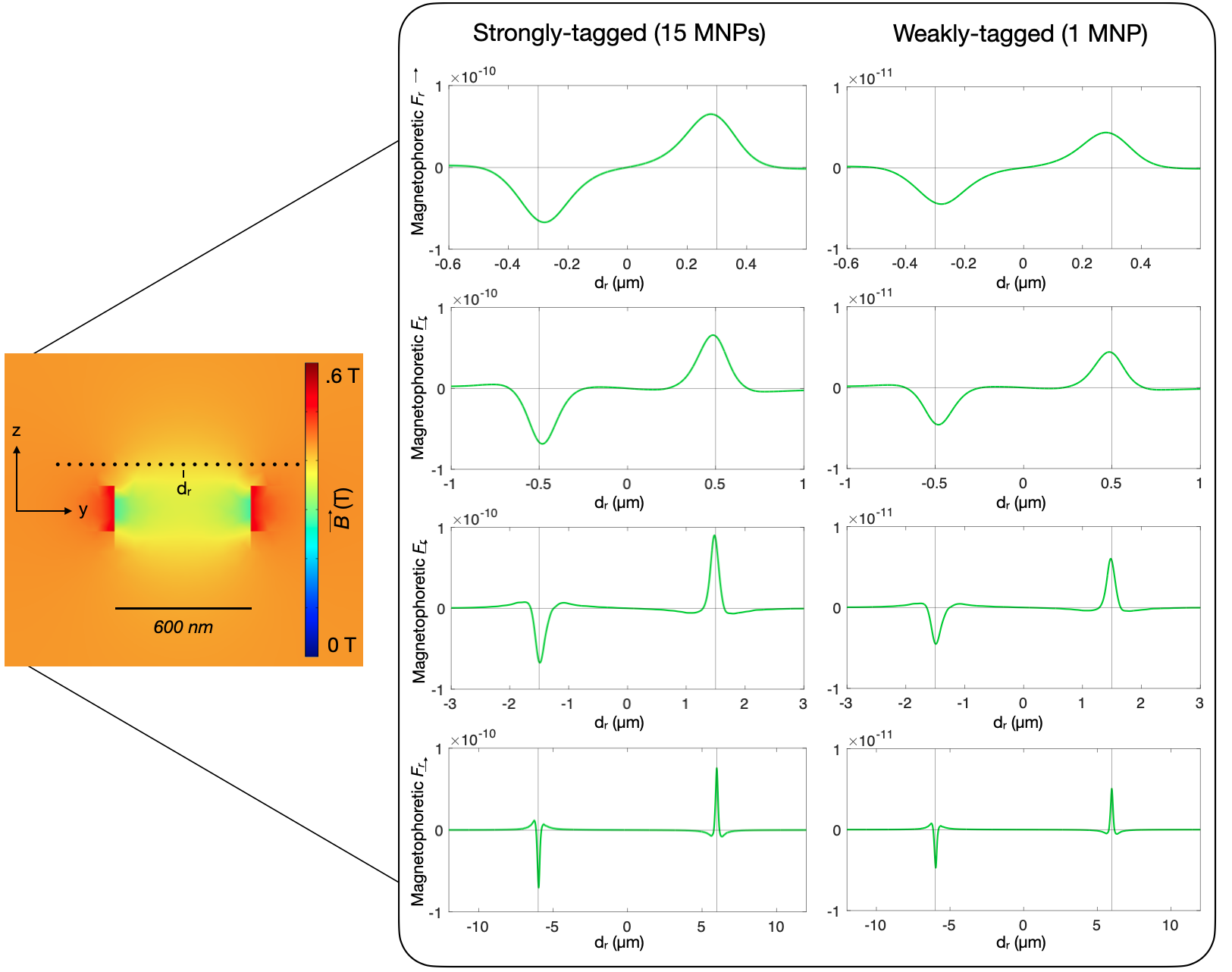


SI fig. 2: Simulations of magnetophoretic versus drag force in the Z-direction for four different pore diameters. All linecuts are taken extending 100 nm above and below the metal coating atop a pore at radius 100 nm from pore edge.
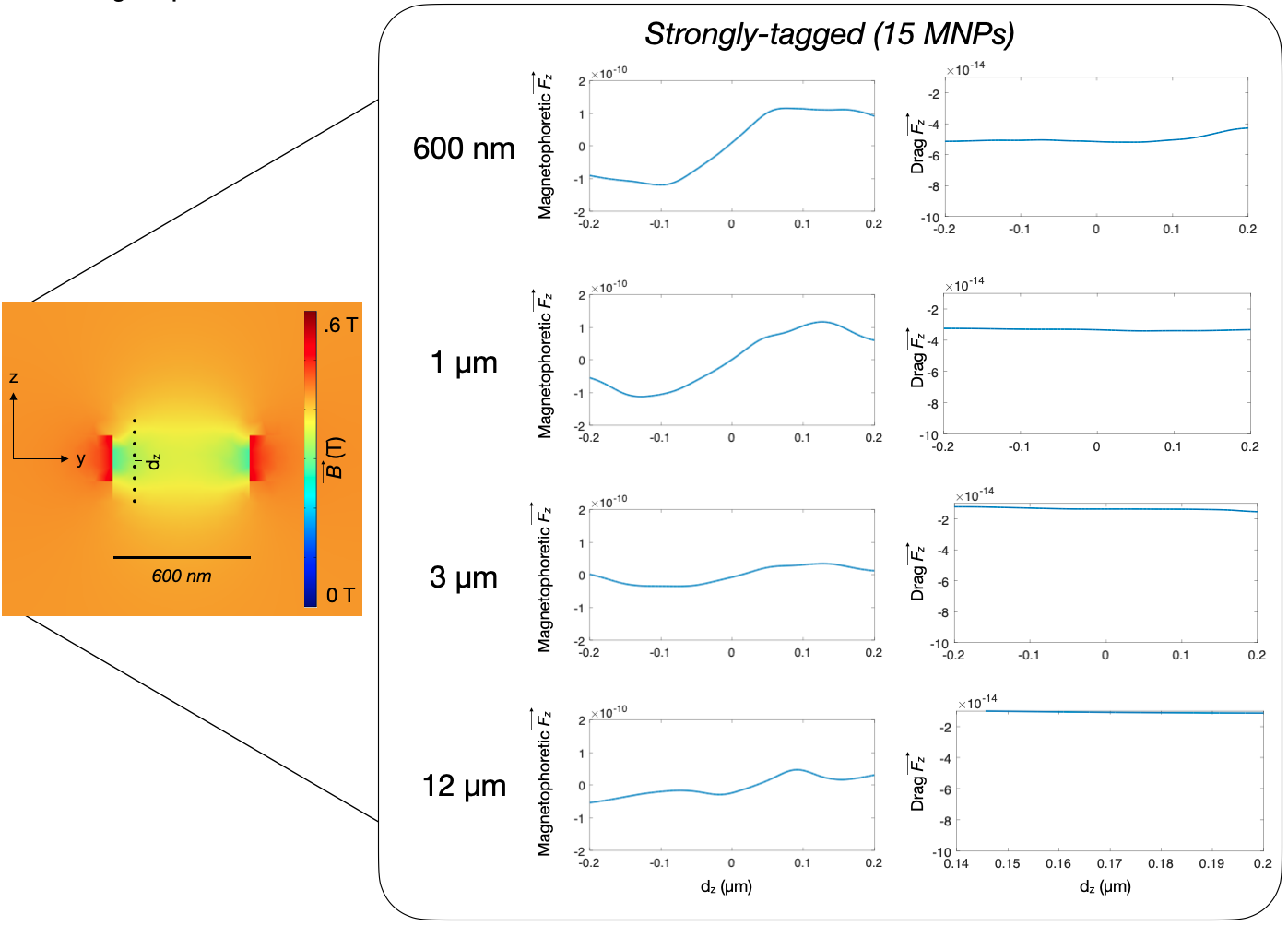


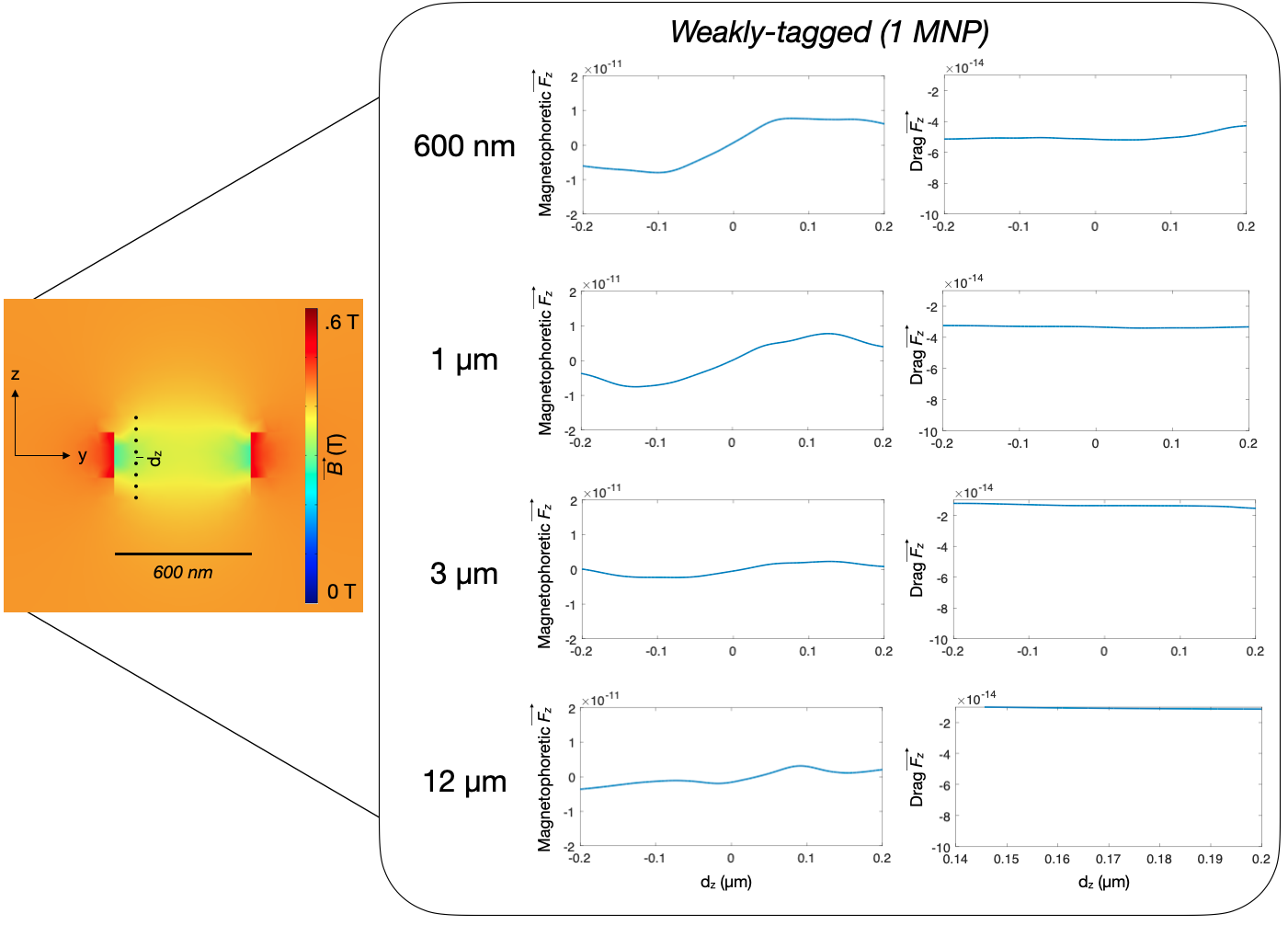


SI fig. 3: Iterative simulations of EVs flowing through a *d* = 3 µm pore at *ɸ* = 2.5 mL/hr; starting grid for pore positions was widened (grid side length 7 µm) compared to simulation in pore diameter scan to avoid conditions with zero particles captured or all 100 particles captured, as this would impede the iterative simulation. Simulations use capture positions and numbers from first membrane (membrane number 1); remaining data points are average of n = 10 iterations (error bars on membrane numbers 2 through 5 are standard deviation of 10 iterations). Inset shows trend of separation between strongly-tagged (Rs) versus weakly-tagged (Rw) EVs.
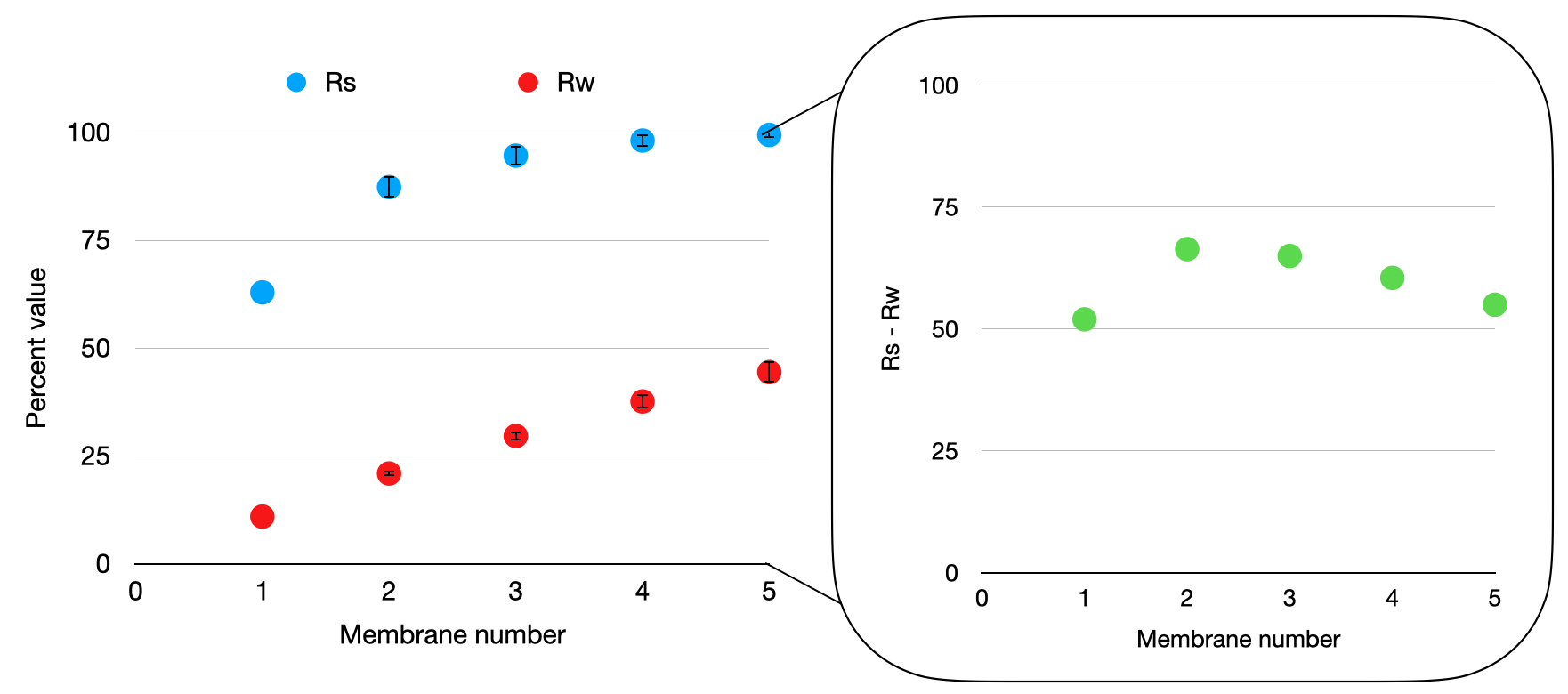


SI fig. 4: Resistance calculated for a single pore versus for flow between pores. Resistance between pores was approximated by treating TENPO as a situation in which fluid flow was flowing in a rectangular channel (defined by the stacked track-etch magnetic membranes) with a height of 100 microns between the two membranes and a channel width and height of 10 microns as an approximate distance between the two pores. Resistance for a single cylindrical pore was quantified for a pore diameter of 3 µm and a pore length of 5 µm.
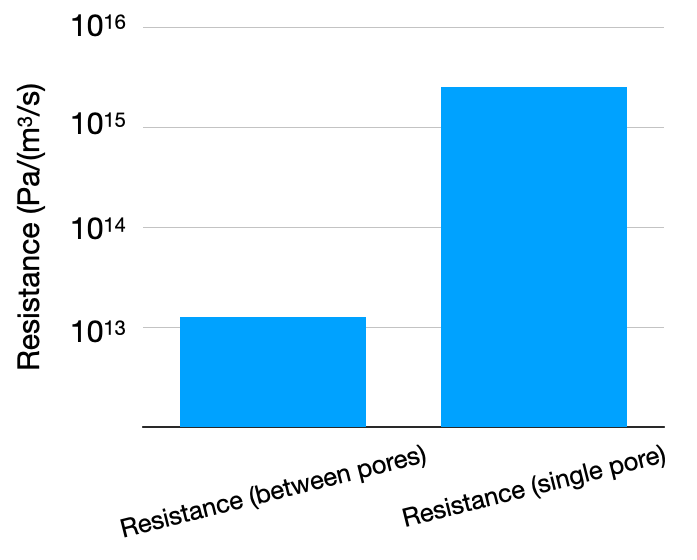


SI fig. 5: Finite-element simulation of sequentially-increasing numbers of clogged pores on-chip; heatmaps at left show example cases of unclogged versus clogged pore grids, while inset graph at right shows impact on central pore drag force for an example simulation in which increasing numbers of pores adjacent to the central pore in the grid are clogged.
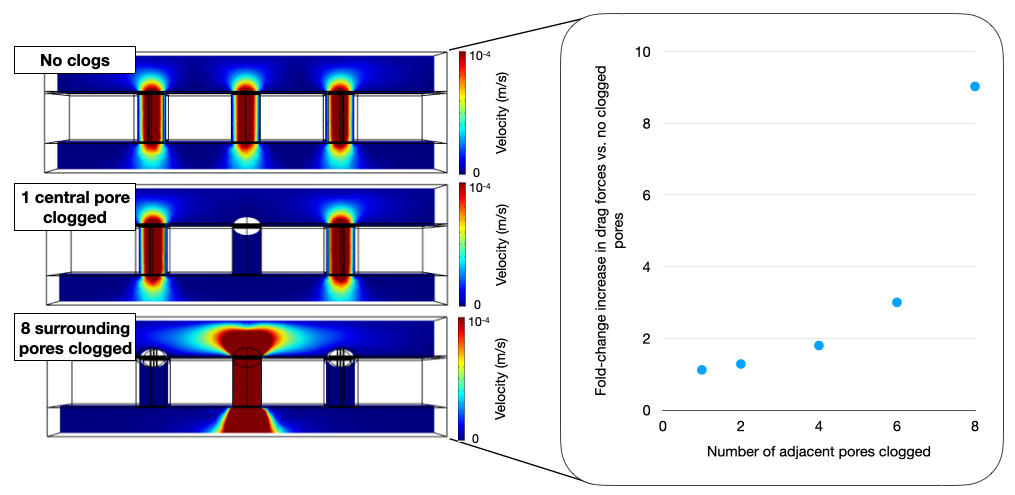


SI fig. 6: Simulation of laminar flow through a *d* = 3 µm pore subjected to clogs of increasing radius. Maximum flow velocity is plotted below the color heatmaps of velocity magnitude; heatmaps of velocity magnitude are shown below.
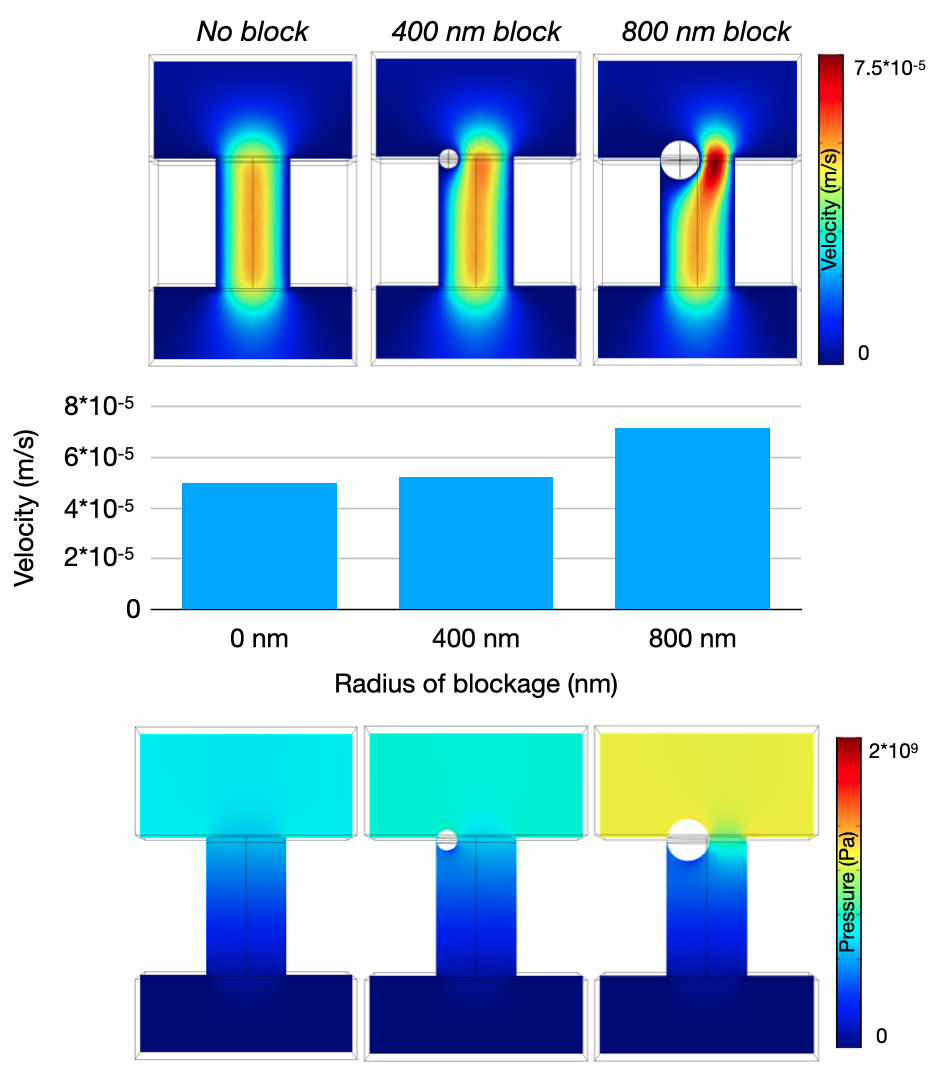


SI fig. 7: Particle tracking simulations of a *d* = 3 µm pore challenged with 100 EV-MNPs at different blockage conditions at a flow rate of *ɸ =* 2.5 mL/hr. Left inset shows particle trajectories for both strongly-tagged and weakly-tagged EVs (reflected EV-MNPs are considered as captured); right graph shows Rs and 1 - Rw for pore clog radii.
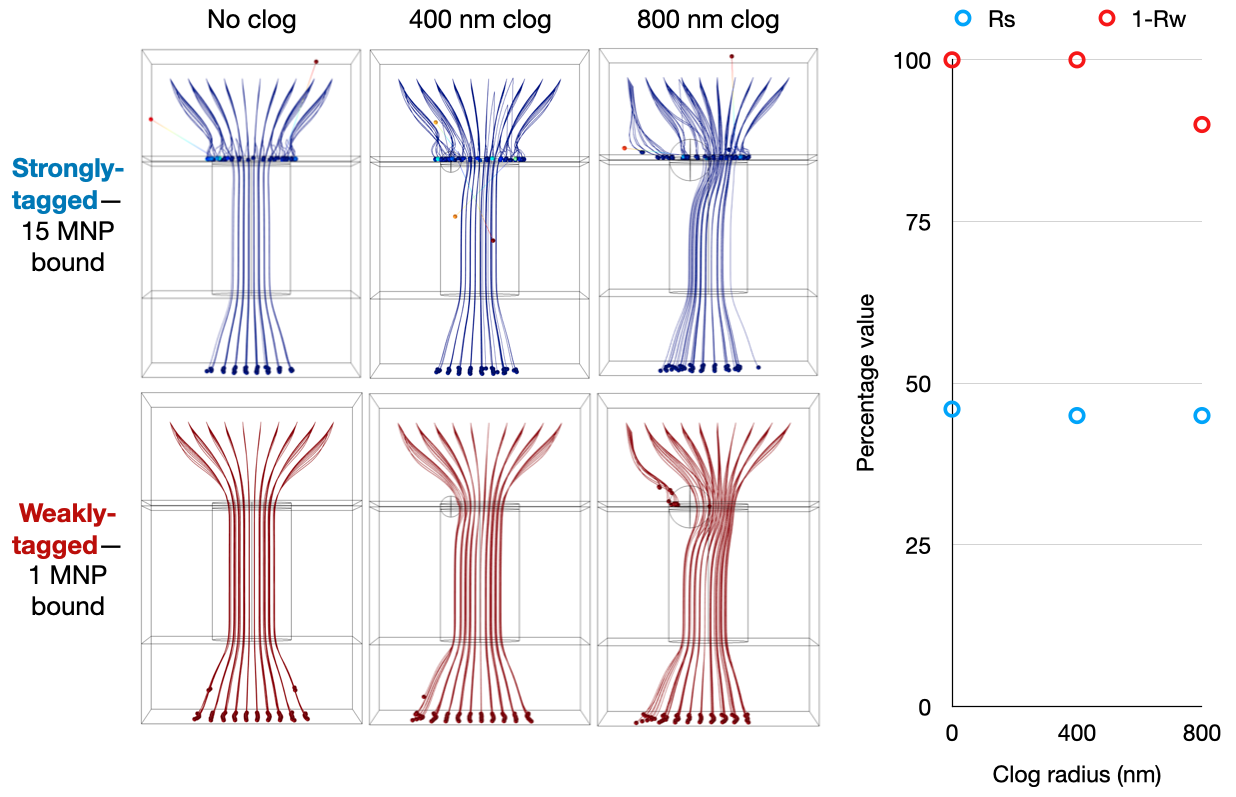


SI fig. 8: Experiment comparing device performance with two different washing flow rate speeds; error bars are from n = 2 PCR replicates on-plate
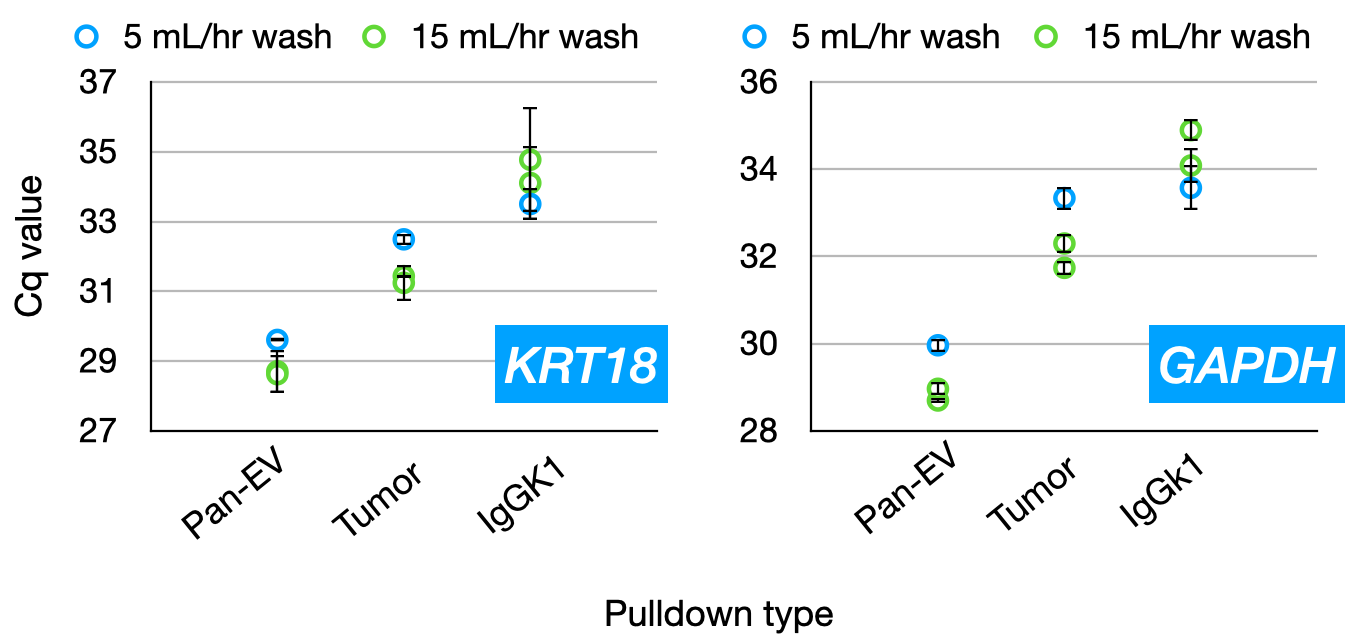
.

SI fig. 9: Experiment comparing device performance with two different magnetic field strengths; error bars are from n = 2 PCR replicates on-plate.
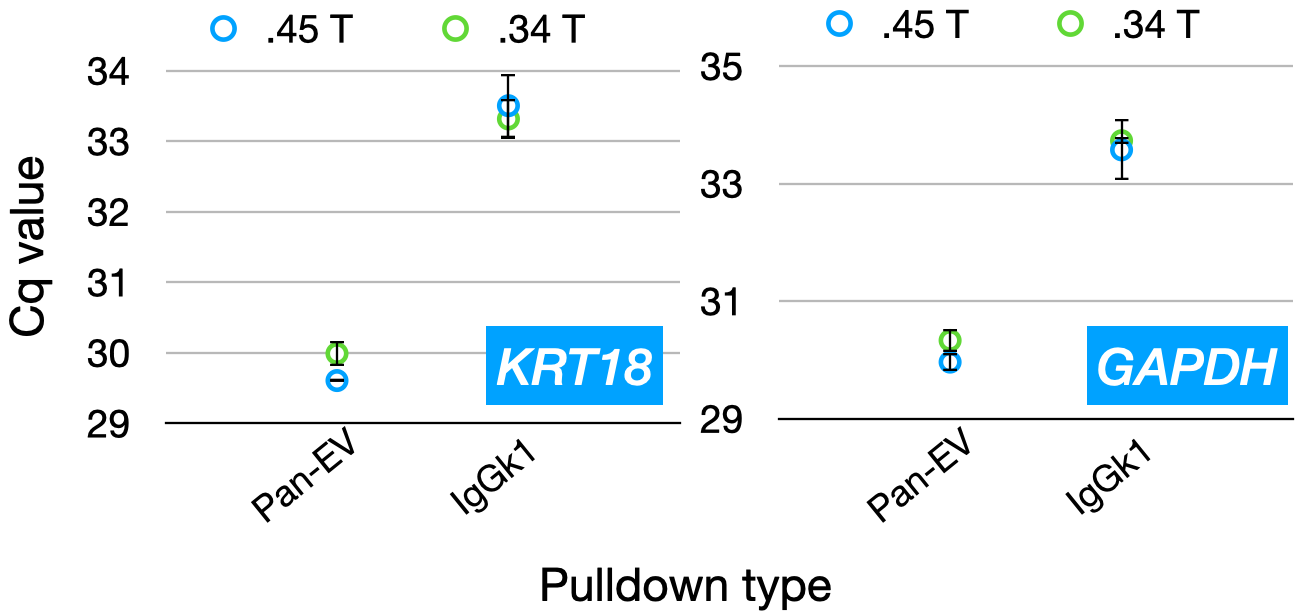


SI fig. 10: Titration curves of different EV amounts of cell culture media into a constant amount of background human plasma (133 µL) at different concentrations isolated via ultracentrifugation and then lysed for nucleic acid analysis via PCR.
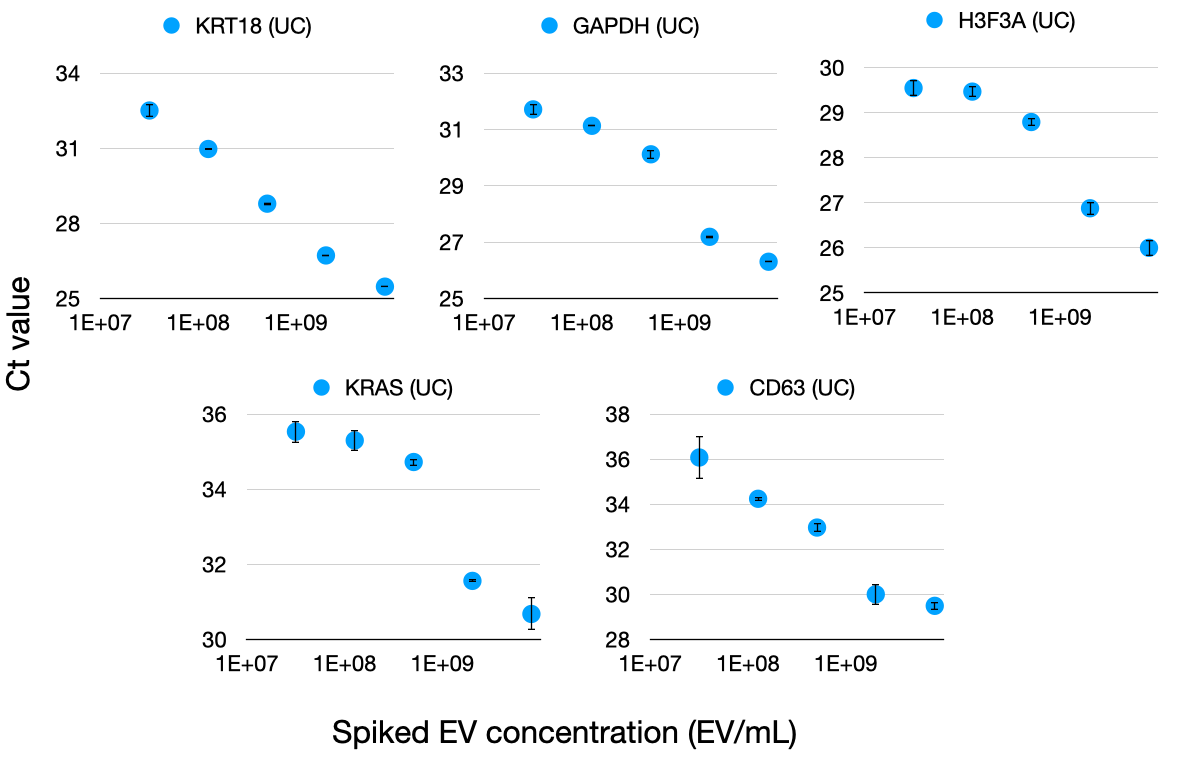


SI fig. 11: PCR data for CD63 for parameter scans shown in figure 3.


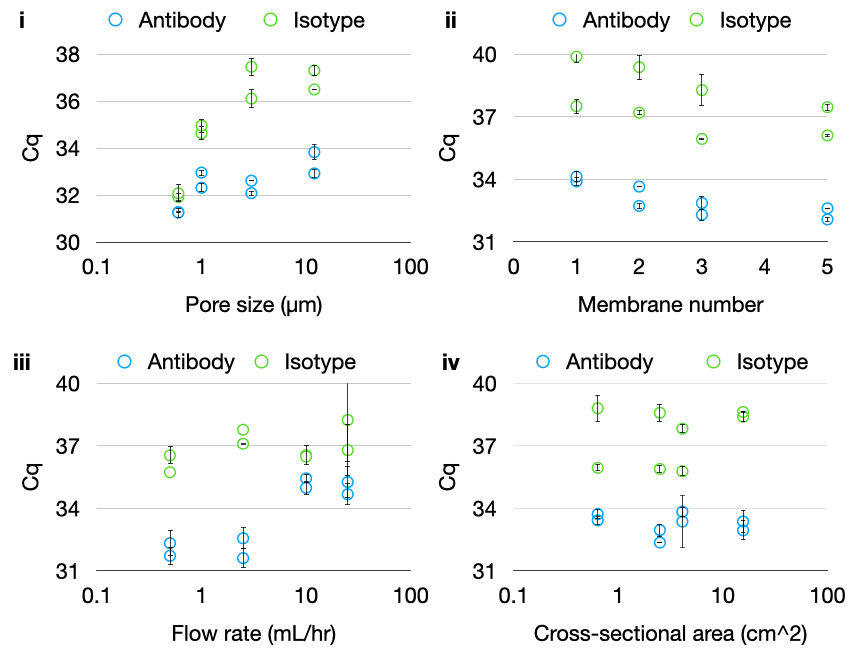


SI fig. 12: Comparison of pore diameters with flow rates scaled by total open porous area on-chip. The *d* = 600 nm devices were run at a slower flow rate (*ɸ =* 1.43 mL/hr) and wash rate (8.60 mL/hr) to account for their decreased cross-sectional porous open area compared to the *d* = 3 µm devices (*ɸ =* 2.5 mL/hr, wash rate = 15 mL/hr).


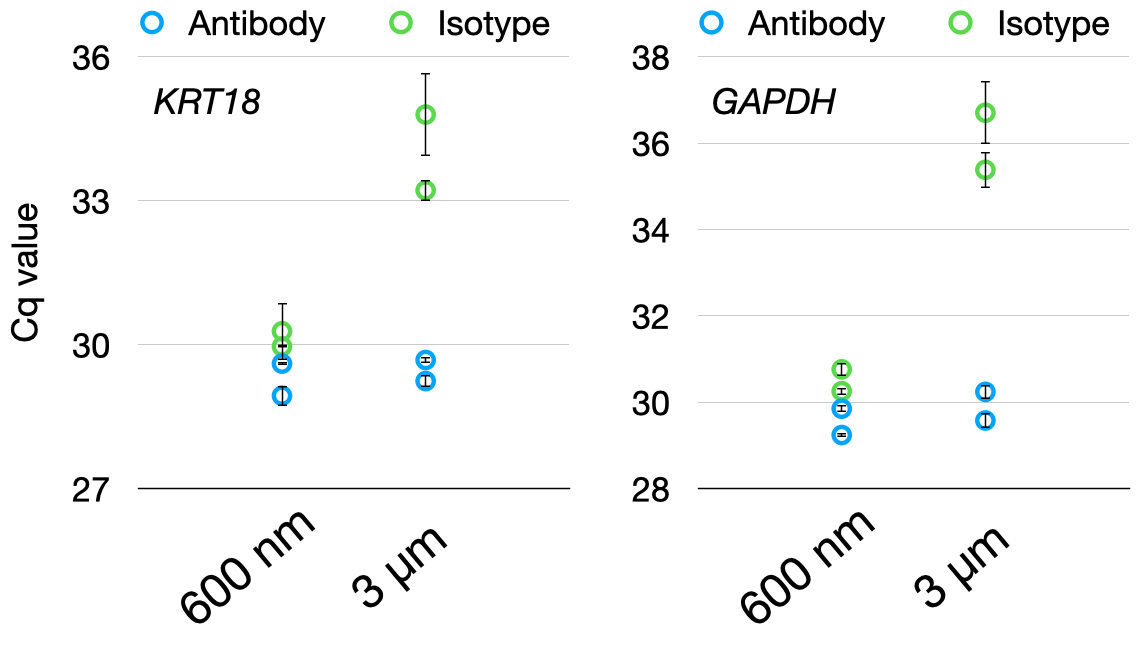


SI fig. 13: Design file patterns for all four cross-sectional area profile devices. For each size, the upper strip represents pieces cut using double-sticky tape, while the lower strip represents pieces cut via Mylar.


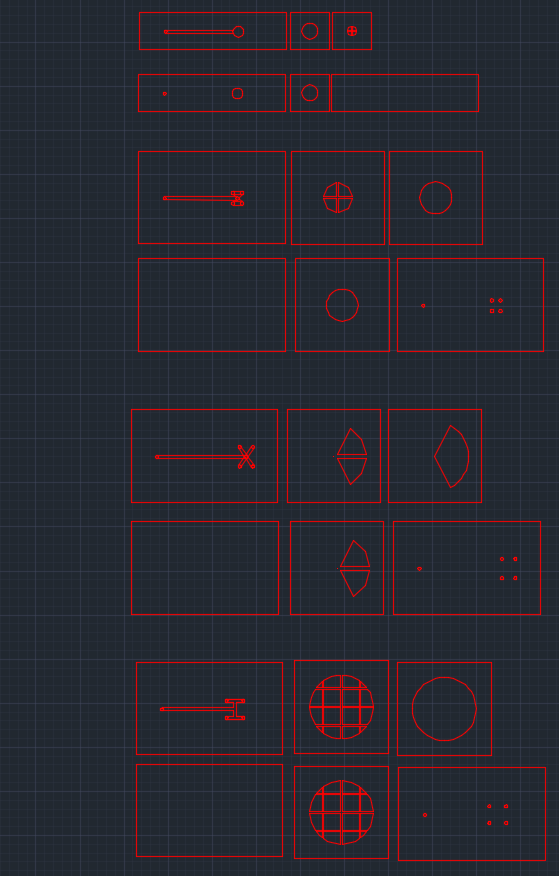


.64 cm^2^

2.5 cm^2^

4.1 cm^2^

15.6 cm^2^

SI fig. 14: Experiment comparing relative enrichment (∆Ct) between antibody vs. isotype for different marker combinations of pan-EV antibody markers. Error bars at left from n = 2 qPCR replicates on-plate, at right from n = 2 device replicates. * = p < .05, ** = p < .01, *** = p < .001 for a two-sample two-tail unequal-variance t-test.


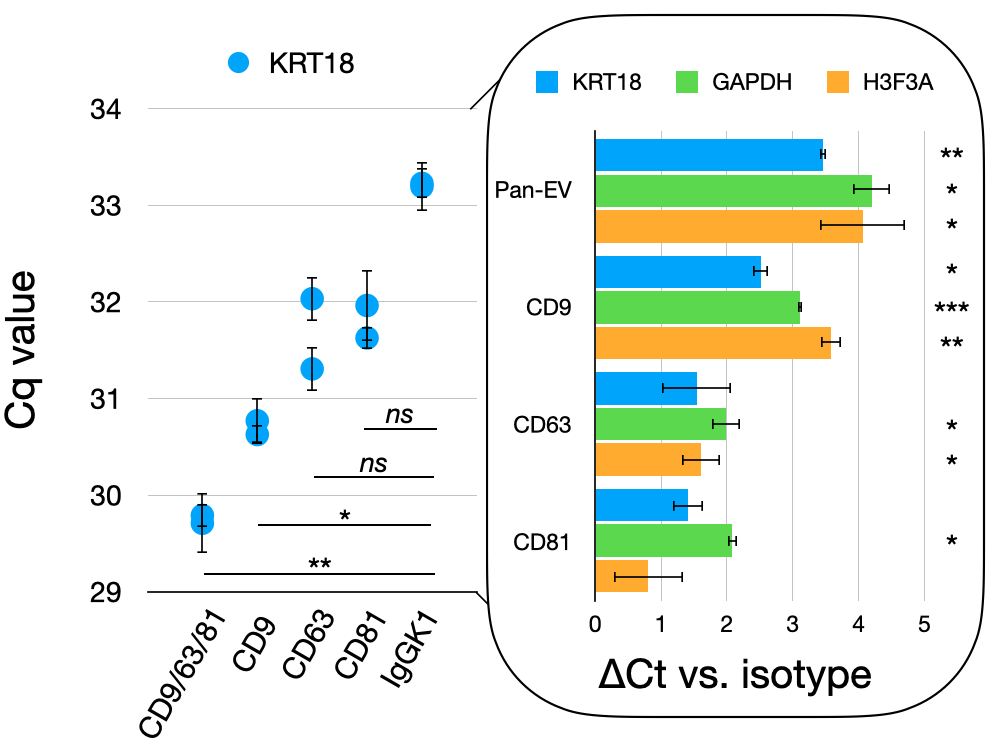


SI fig. 15: Whole-EV ELISA comparing relative expression of pan-EV surface markers on EVs from pancreatic cancer cell culture media. Error bars from n = 3 technical replicates on-plate.

SI fig. 16: Comparison of run-to-run variation in device performance versus isotype for pan-EV antibodies in pancreatic cancer cell culture media spiked into FBS. Data is summarized from three runs of two replicates each (two antibody, two isotype) run on different days and characterized for three different markers (KRT18, GAPDH, H3F3A). Graph of average raw Cq values for six antibody-labeled versus six isotype-labeled devices across three different runs. Error bars are from average of six device replicates.


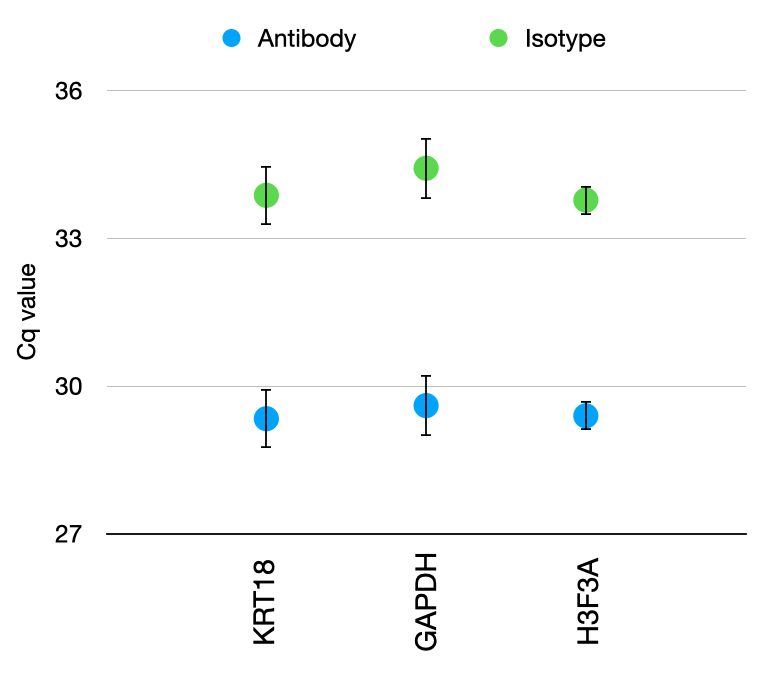


SI fig. 17: Comparison of TENPO vs. UC for total EVs captured (NTA), albumin depletion (ELISA), and protein recovery. Albumin depletion was calculated relative to a literature-derived value for albumin concentration in human plasma of 4 g/dL [52]; at a dilution of 1:500,000, the original input plasma (Zen-Bio) yielded a fluorescence result well beyond the logistic calibration curve limit of 1200 ng/mL of the ELISA. Error estimates are from duplicate measurements.

|  | Total particles per mL | Albumin depletion from 1 mL plasma | Total protein recovered (µg/mL) |
| --- | --- | --- | --- |
| TENPO | **6.60*10^9^** +/- 1.41*10^9^ | **36.33x** | **205.5 +/- 7.8** |
| UC | **1.14*10^10^** +/- 3.68*10^9^ | **4.50x** | **216.0 +/- 4.2** |

SI fig. 18: Comparison of size ranges of EVs isolated via TENPO vs. UC using NTA.
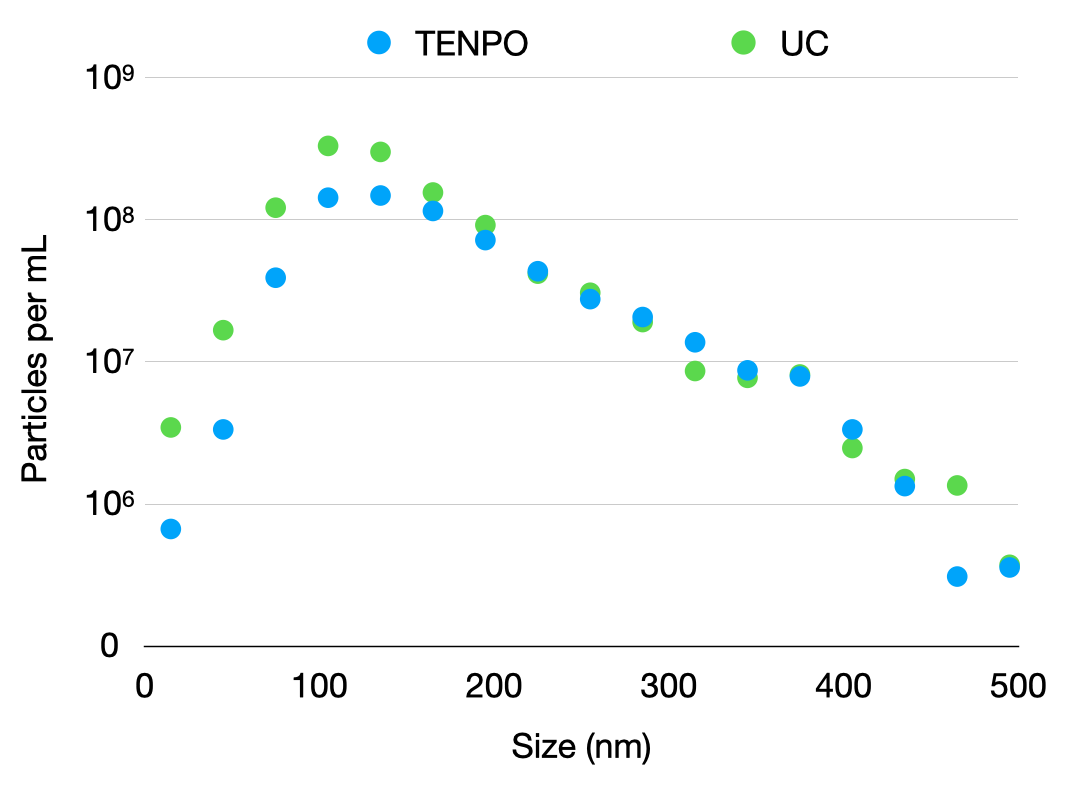


SI fig. 19: Cancer versus healthy spike-in data for pancreatic, liver, and lung cancer tumor pulldowns.


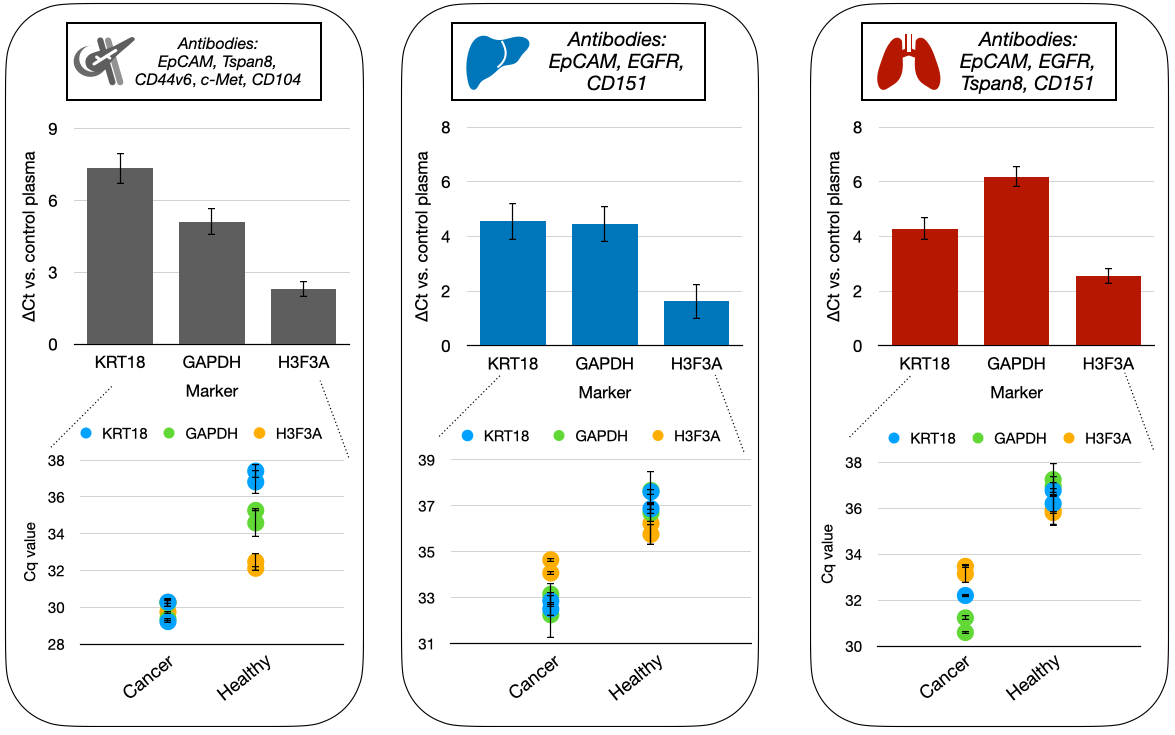


SI fig. 20: Calibration curve for commercial albumin ELISA (Thermo Fisher). Calculated via [51].


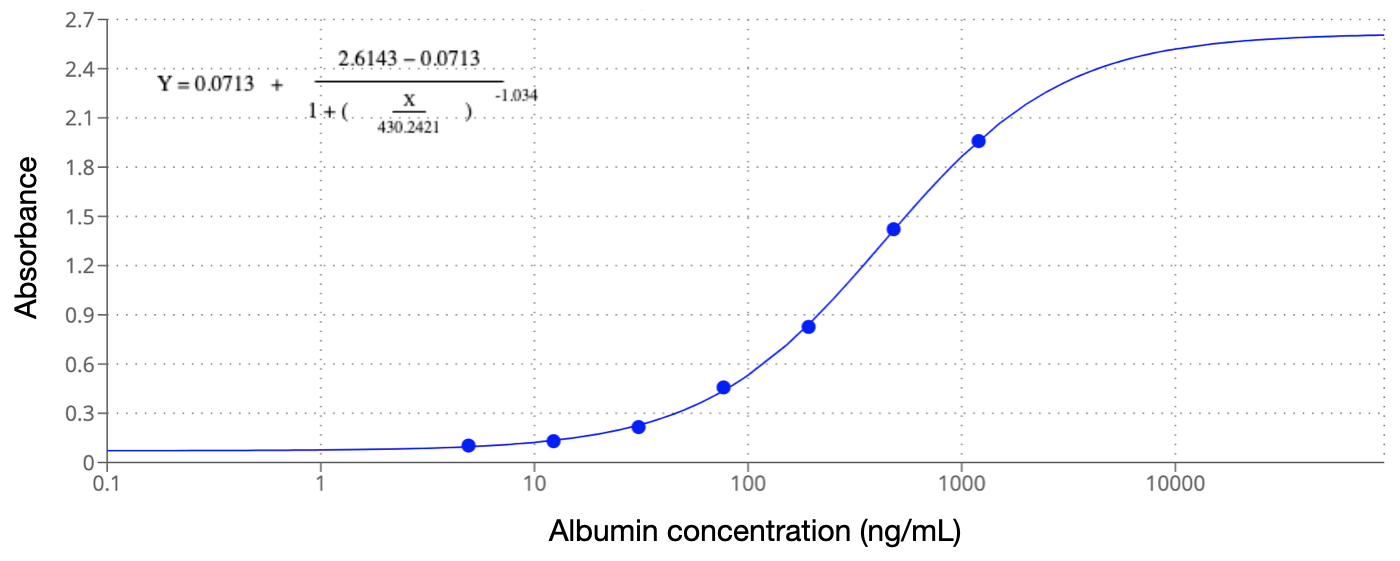

Supplement: Supplementary file 1 — Supplementary Figures. [file 41598_2023_39746_MOESM1_ESM.docx]
